# Carboplatin restricts peste des petits ruminants virus replication by suppressing the STING-mediated autophagy

**DOI:** 10.3389/fvets.2024.1383927

**Published:** 2024-05-15

**Authors:** Rui Zhang, Zhanying Hu, Dingcheng Wei, Ruizhe Li, Yanmin Li, Zhidong Zhang

**Affiliations:** College of Animal and Veterinary Sciences, Southwest Minzu University, Chengdu, Sichuan, China

**Keywords:** peste des petits ruminants virus, Carboplatin, antiviral, STING, autophagy

## Abstract

Peste des petits ruminants virus (PPRV) is a morbillivirus that causes the acute and highly pathogenic infectious disease peste des petits ruminants (PPR) in small ruminants and poses a major threat to the goat and sheep industries. Currently, there is no effective treatment for PPRV infection. Here, we propose Carboplatin, a platinum-based regimen designed to treat a range of malignancies, as a potential antiviral agent. We showed that Carboplatin exhibits significant antiviral activity against PPRV in a cell culture model. The mechanism of action of Carboplatin against PPRV is mainly attributed to its ability to block STING mediated autophagy. Together, our study supports the discovery of Carboplatin as an antiviral against PPRV and potentially other closely related viruses, sheds light on its mode of action, and establishes STING as a valid and attractive target to counteract viral infection.

## Introduction

Peste des petits ruminants (PPR) is one of the World Organization for Animal Health (WOAH) listed notifiable transboundary viral diseases of domestic and wild small ruminants. Of particular note, in goats and sheep that is associated with high morbidity and mortality (1). Mortality can be as high as 100%, and it is estimated that the economic losses caused by PPR were approximately USD$2.9 million per year during 2012–2017 (2, 3). Clinically, the disease is characterized by pyrexia, erosive stomatitis, pneumonia and diarrhea (4, 5). Importantly, PPR often causes fetal mummification, abortions late in pregnancy, or the birth of dead or weak lambs that die within a few days (6–8). Since the first report of PPR by Gargadennec and Lalanne in Côte d’Ivoire in 1942, the disease has spread so alarmingly that its geographical distribution has expanded through over 70 countries in Africa, the Middle and Near East, South Asia, and China (9–11). Outbreaks of PPR in Georgia (2016) and Bulgaria (2018) have been reported, posing a serious threat to Europe (12). Currently, about 80% of world’s sheep and goat populations are threatened by PPR (11, 13). As one of the most wide-spread and devastating infectious disease, the huge impact of PPR on small ruminant production has led the United Nations Food and Agriculture Organization (FAO) and WOAH to propose and initiate the PPR Global Control and Eradication Strategy (PPR GCES), with the purpose of eradicating the disease by 2030 (14).

Peste des petits ruminants virus (PPRV) is an enveloped ribonucleic acid (RNA) with a non-segmented genome of negative sense within the genus *Morbillivirus* in the family *Paramyxoviridae* (15). It is in the same group as measles virus (MeV), rinderpest virus (RPV), canine distemper virus (CDV), phocine distemper virus (PDV) and dolphin morbillivirus (DMV) (15, 16). The PPRV genome is 15,948 nucleotides in length (16) and encodes six structural proteins in sequential order: the nucleocapsid (N) protein, the phospho (P) protein, the matrix (M) protein, the fusion (F) protein, the hemagglutinin (H) protein, and the large (L) protein. As with other morbilliviruses, the P gene of PPRV produces two nonstructural proteins, C and V (17). Based on N or F gene sequences, the virus has been divided into four lineages, although it has a signal serotype (15, 16, 18). Upon infection, PPRV localizes to and replicates in the tonsils and lymph nodes, causing severe lymphocytolysis in lymphoid tissues and a subsequent immunodeficiency to lymphoid depletion (19–21). The pathogenesis of PPRV infection is characterized by the induction of strong but transient immunosuppression of host protective responses, which leads to increased susceptibility to opportunistic infections that affect the outcome of the infection (19, 21–23). Therefore, it is particularly important to prevent PPRV infection and replication for successful control of the disease.

Currently, no antiviral drug has been approved for therapeutic application to control PPRV infection. Vaccination is the main method available for the effective prevention and control of PPR. PPRV Nigeria75/1 (lineage II) and PPRV Sungri 96 (lineage IV) are currently the most widely used live attenuated vaccines, and their efficiency has been most extensively tested and validated (13, 24–27). However, these live attenuated vaccines are heat-sensitive in subtropical climates and have high production costs (13). Thus, there is an urgent need to develop safe and effective antiviral agents against PPRV.

Carboplatin (cis-diammine-1,1-cyclobutane decarboxylate platinum [II]) is a second-generation platinum-based chemotherapeutic agent that has been extensively utilized in the clinic to treat a range of malignancies in humans (28–31) and has been reported as being the safest platinum derivative to be used in pregnancy (31, 32). Following cellular uptake, Carboplatin binds covalently to DNA nucleobases and cross-links DNA to form a variety of DNA adducts and induces apoptosis through the inhibition of tumor cell apoptosis and other mechanisms, resulting in a pro-inflammatory, antitumor immune response (33, 34). Studies have demonstrated the clinical efficacy of Carboplatin in the treatment of canine appendicular osteosarcoma and feline oral and cutaneous squamous cell carcinomas, validating that Carboplatin is a useful anticancer agent for dogs and cats with solid tumors (28, 35, 36). It has been suggested that the platinum-containing compound Carboplatin plays a role in stimulating immune responses against tumors (36, 37). What is more, in the study by Chen et al., they found that the platinum-based chemotherapeutic agent can modulate viral replication in patients receiving chemotherapy (38). Therefore, it is reasonable to explore the potential influence of Carboplatin in the infection of various viral diseases in humans and animals.

Autophagy is an evolutionarily conserved pathway for the degradation of unnecessary or dysfunctional intracellular organelles, unfolded or misfolded proteins, and pathogenic microorganisms to maintain cellular homeostasis in response to a variety of stresses (39, 40). A large body of evidences have demonstrated that virus replication and autophagy are mechanistically interconnected and autophagy plays a dual role during viral infections (41). Mounting studies have suggest that there are complicated interconnections between the viral replication process and autophagy (42) and many viruses have evolved a range of strategies to exploit autophagy for its replication (39, 41). It has been recently demonstrated that the platinum-based chemotherapeutic agent can promote hepatitis B virus (HBV) replication via inducing autophagy (38). A growing number of studies have shown that PPRV can hijack autophagy to facilitate its replication (8, 43–46). However, the effects of Carboplatin on morbilliviruses replication and the association between Carboplatin and autophagy have not yet been investigated.

This study aimed to determine the effect of Carboplatin on PPRV replication *in vitro* and systematically investigate its mechanism of action. Our data will elucidate the potential molecular mechanisms of Carboplatin upon viral infection and might provide further insights into the development of novel promising strategies for the control of acute PPRV infection, as well as for a possible application to other closely genetically related pathogens such as MeV.

## Materials and methods

### Cell culture and virus propagation

African green monkey kidney (Vero) cells (ATCC: CCL-81) were cultured in Dulbecco’s modified Eagle’s medium (DMEM, Gibco) supplemented with 10% heat-inactivated fetal bovine serum (FBS, Gibco) and penicillin–streptomycin solution (100 U/mL and 100 μg/mL, respectively; Gibco) as monolayers in cell culture flasks or dishes at 37°C in a humidified atmosphere of 5% CO_2_ in air.

The PPRV attenuated vaccine strain Nigeria 75/1 was obtained from our laboratory’s culture collection. A viral stock was generated by infecting monolayers of Vero cells. PPRV was inoculated into Vero cells at a multiplicity of infection (MOI) of 10 and cultured in DMEM supplemented with 2% FBS at 37°C with 5% CO_2_ for 6 days until a pronounced cytopathic effect (CPE) was observed in approximately 80% of the cells. The virus-containing media were collected, and the cells were lysed by two freeze–thaw cycles. The supernatant and cell lysate were combined, centrifuged at 4500 g for 10 min to remove cell debris, filtered (0.45 μm), aliquoted, and stored at −80°C.

### Cytotoxicity assay

Cytotoxicity of the compounds was assessed using the CCK-8 assay. Monolayers of Vero cells were seeded in a 96-well plate and incubated for 24 h. Then, the medium was replaced with 100 μL of culture medium supplemented with different concentrations of Carboplatin (0, 20, 40, 60, 80, or 100 μM; Selleck, S1215) and incubated for 24 h at 37°C with 5% CO_2_. Next, 10 μL of CCK-8 stock solution was added to each well and incubated at 37°C for 2 h. Finally, the optical density (OD) of the quadruplicate wells at 450 nm was determined using an ELISA microplate reader (PerkinElmer, VICTOR Nivo^™^, United States).

### Pre-infection assay with Carboplatin

Monolayers of Vero cells were seeded in 6-well plates and treated with Carboplatin for 24 h. On the day of infection, cells were infected with PPRV (1 MOI) for 1 h at 37°C. After that, the cells were washed twice with PBS before being treated with Carboplatin dissolved in DMEM supplemented with 2% FBS. Six different concentrations of Carboplatin (0, 20, 40, 60, 80, or 100 μM) were set to determine the dose with the most significant effect on PPRV replication. At 48 h post-infection (hpi), the supernatant was harvested from infected cell cultures and the viral titer was determined using the TCID50 assay, and cells were harvested and viral mRNA levels in cell lysates were determined using quantitative real-time PCR.

### Viral titration *in vitro*

To determine virus titers, virus suspensions were prepared by 10-fold serial dilution of the virus stock, collected from PPRV-infected and 100 μM Carboplatin pre-treated, PPRV-infected cells, in DMEM without supplements. Monolayers of Vero cells were seeded in a 96-well plate the day before the titration. Vero cells were inoculated on the day of infection with 100 μL of the supernatant for 1 h at 37°C. Cells were then washed twice with PBS, before being overlaid with DMEM containing 2% FBS and incubated at 37°C for 6–7 days. Viral titers were assessed using the Reed and Muench method (47) and expressed as 50% tissue culture infective dose (TCID_50_) /mL.

### Transmission electron microscopy

Vero cells treated or untreated with 100 μM Carboplatin were infected with PPRV Nigeria 75/1 (MOI 1) for 48 h. After infection for 48 h, cells were scraped and harvested by centrifugation at 10,000 rpm for 5 min. The cells were fixed in 2.5% glutaraldehyde overnight at 4°C, washed three times with PBS, and then post-fixed with 1% osmium tetroxide for 3 h at 4°C with shaking. After three washes with PBS, the samples were dehydrated in a series of graded ethanol solutions and embedded in Spurr’s plastic resin. The cells were polymerized overnight at 70°C in a drying oven. Ultrathin sections (70 nm) were prepared using an ultramicrotome (Ultracut R, Leica, Germany).

### Western blotting and antibodies

Cells treated or untreated with 100 μM Carboplatin were harvested and washed with cold PBS at 48 h after PPRV infection, then lysed in RIPA lysis buffer containing protease and phosphatase inhibitor-containing RIPA lysis buffer (50 mM Tris [pH 7.4], 150 mM NaCl, 1% Triton X-100, 1% sodium deoxycholate, 0.1% SDS; Beyotime catalog number P0013B) supplemented with 1% 100 mM phenylmethylsulfonyl fluoride. The whole-cell extracts were clarified by centrifugation at 16,000 rpm for 10 min at 4°C. Protein concentrations were determined using a bicinchoninic acid protein assay kit (23,225; Thermo Scientific). Protein samples were denatured in equivalent 2 × SDS-PAGE sample buffer (S3401; Sigma-Aldrich) by heating for 5 min at 95°C. Proteins were separated by 10% polyacrylamide gel electrophoresis and then transferred to polyvinylidene fluoride membranes (Millipore, ISEQ00010) at 200 mA for 2 h. Membranes were blocked with 5% nonfat milk powder in Tris-buffered saline with Tween 20 (TBST) for 2 h at room temperature (RT) and then incubated with primary antibodies overnight at 4°C. After washing three times with TBST, the membranes were incubated with peroxidase-conjugated secondary antibodies for 1 h at RT. Bound antibodies were visualized using Clarity Western ECL substrate (Bio-Rad). A mouse horseradish peroxidase-coupled monoclonal antibody specific to β-tubulin (Proteintech, 66,240-1-Ig) was used as a loading control. Bands were detected using a GE Healthcare Amersham Imager 600 in the automatic exposure mode to ensure that the bands were not saturated.

Rabbit polyclonal antibodies anti-STING (D1V5L; catalog number 50494S) and anti-LC3B (catalog number 2775 s) were purchased from Cell Signaling Technology. Mouse monoclonal anti-β-tubulin antibody (catalog number 66240-1-Ig), rabbit polyclonal antibodies anti-XBP1 (catalog number 24864-1-AP), anti-EIF2S1 (catalog number 11170-1-AP), and anti-PERK (catalog number 24390-1-AP) were obtained from Proteintech. Rabbit polyclonal antibodies anti-ATF6 (catalog number ab37149), anti-phospho-EIF2S1 (E90; Ser51; catalog number ab32157) were from Abcam. Rabbit polyclonal antibody anti-phospho-PERK (Thr981; catalog number sc-32577) was from Santa Cruz Biotechnology. Rabbit polyclonal antibody anti-ATG5 (catalog number NB110–53818) was obtained from Novus Biologicals. Mouse monoclonal antibodies against the N protein of PPRV were obtained from the Lanzhou Veterinary Research Institute, Chinese Academy of Agricultural Sciences.

### Quantitative real-time PCR

Cells treated or untreated with 100 μM Carboplatin were harvested at 48 h after PPRV infection, and total RNA was extracted using the RNeasy Plus Universal Mini Kit (Qiagen catalog number 73404) according to the manufacturer’s protocol. First-strand cDNA was synthesized using the Maxima H Minus cDNA synthesis master mix with dsDNase (Thermo Scientific, M1682). PowerUp SYBR Green Master Mix (Applied Biosystems, 1,801,040) was used to perform the quantitative real-time PCR, and the thermal cycling conditions were set according to the manufacturer’s instructions. Live-attenuated vaccine PPRV Nigeria75/1 was used as a positive control for real-time PCR. The primers used for qPCR were as follows: PPRV forward, 5′-AGAGTTCAATATGTTRTTAGCCTCCAT-3′; PPRV reverse, 5′-TTCCCCARTCA CTCTYCTT TGT-3′; GAPDH forward, 5′-CGAGATCCCTCCAAAATCAA-3′; GAPDH reverse, 5′-TGAC GATCTTGAGGCTGTTG-3′.

### RNA interference

To construct lentiviral shRNA vectors, shRNAs were designed using BLOCK-iTRNAi Designer (Invitrogen). The shRNAs used in this study were synthesized by Sangon Biotech (Shanghai, China) and cloned into a pLKO.1-TRC cloning vector, following a standard protocol. Vero cells cultivated in 6-well cell culture plates were transfected with RNA interference oligonucleotides using Lipofectamine 3000 (Invitrogen) according to the manufacturer’s instructions. The target sequences were as follows: STING, 5′- GCATTACAACCACCTGCTACG-3′ and ATG5, 5′-GCTTCGAGATGTGTGGTTTGG-3′.

### Lentivirus packaging and infection

For packaging lentivirus, 1.5 μg psPAX2 packaging plasmid (Addgene, 12,260), 1 μg pMD2.G envelope plasmid (Addgene, 12,259), and 2 μg pLKO.1 plasmid were co-transfected into 4 × 10^6^ Lenti-X 293 T cells using Lipofectamine 3,000 transfection reagent (Thermo, L3000015). The supernatant was collected at 36 hpi, filtered, and stored at −80°C. Vero cells were incubated with the viral particles in the presence of 8 μg/mL polybrene (Solarbio, H8761) for 24 h and treated with 5 μg/mL puromycin (Invitrogen, A1113803) for 3 days. Protein expression levels were determined by western blotting analysis.

### Statistical analysis

Data are expressed as means ± standard deviation (SD). The significance of the variability between the different treatment groups was calculated by two-way analysis of variance (ANOVA) using GraphPad Prism software (version 6.0). Differences were considered statistically significant at *p* < 0.05.

## Results

### Carboplatin inhibits PPRV replication

**Figure 1:**
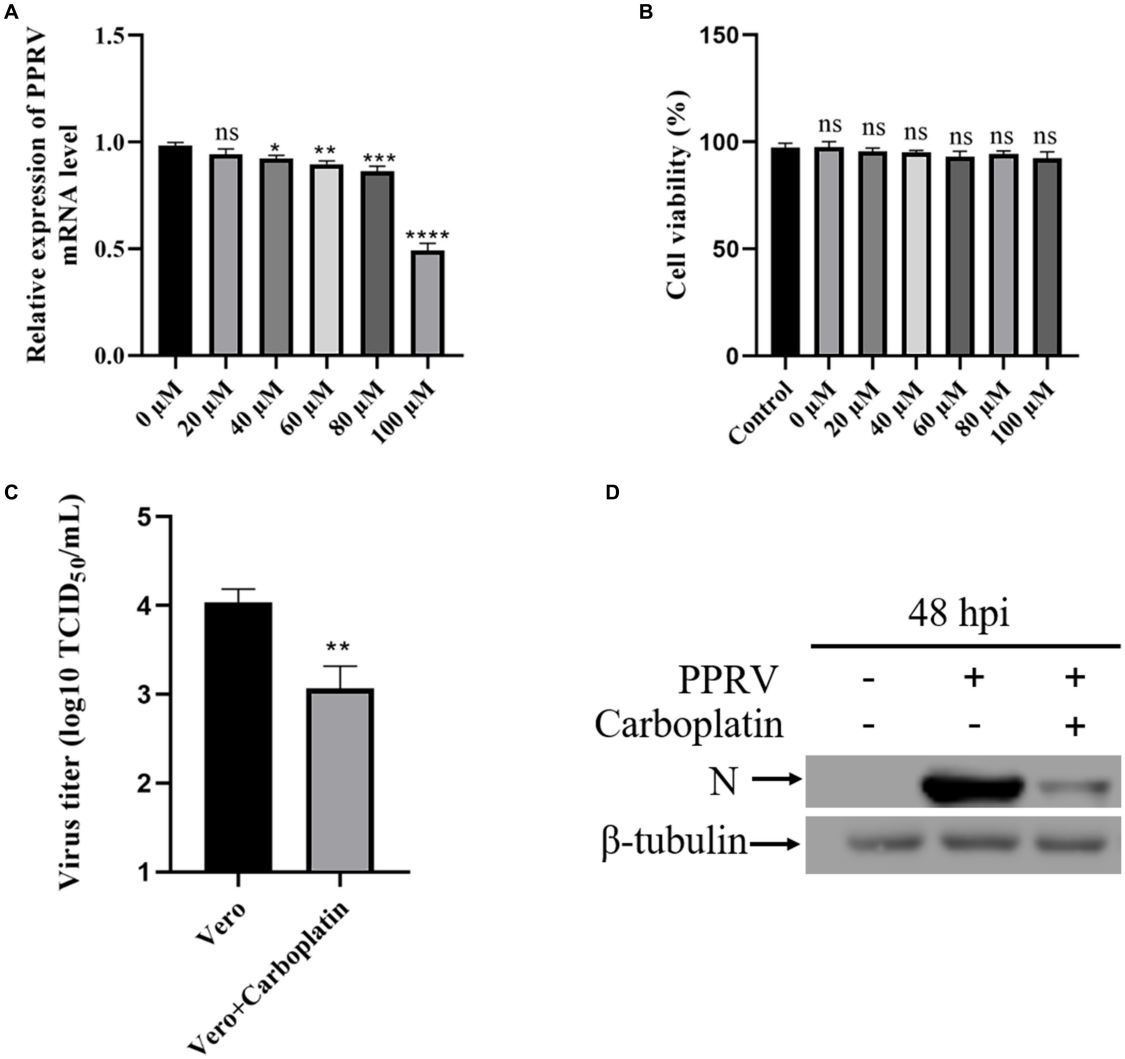
Carboplatin (100 μM) significantly inhibits PPRV replication in Vero cells. **(A)** PPRV mRNA levels in various concentration Carboplatin-treated, PPRV-infected Vero cells (MOI = 1, 48 hpi) were measured by qPCR. The data show the means ± SD; *n* = 3; *p < 0.005; **p < 0.01; ***p < 0.001; ****p < 0.0001. **(B)** Cytotoxicity of different concentration of Carboplatin. Viability was normalized to non-treated control. The data show the means ± SD; *n* = 3; ns, no significance. **(C)** Control and (100 μM) Carboplatin pre-treated cells were infected with PPRV (MOI = 1), and virus titers were measured by TCID_50_ (48 hpi). The data show the mean ± SD; *n* = 3; **p < 0.01. **(D)** Western blotting analysis of PPRV N protein in PPRV-infected wild-type and Carboplatin-treated cells (MOI = 1, 48 hpi). β-tubulin was used as a loading control.

To examine the effect of Carboplatin on PPRV infection, Vero cells were pretreated with different concentrations of Carboplatin for 24 h and subsequently infected with PPRV. 48 h after infection, viral mRNA levels in cell lysates were determined using quantitative real-time PCR. The results showed that Carboplatin pretreatment resulted in a dose-dependent reduction in PPRV replication. A significant reduction in mRNA levels was observed in cells infected with PPRV and treated with Carboplatin at a concentration of 40 μM. In particular, 100 μM Carboplatin led to an approximately 70% reduction in viral mRNA levels when compared to the 0 μM DMEM vehicle control (Figure 1A), indicating that a high concentration of Carboplatin is required for its antiviral effect. Cell viability was not affected by Carboplatin treatment alone (Figure 1B), suggesting that the reduction in viral mRNA levels was not due to cytotoxicity. The inhibitory effect of Carboplatin at 100 μM was also observed in virus yield, as an obvious reduction in viral titer was noted in pretreated cells compared to that in untreated control cells at 48 hpi (Figure 1C). Moreover, in agreement with the TCID_50_ results, western blotting results revealed that viral N protein expression was significantly lower in Carboplatin-treated cells than in untreated PPRV-infected cells (virus-only control; Figure 1D). Taken together, these data suggest that Carboplatin is highly potent in suppressing PPRV replication in Vero cells.

### Carboplatin inhibits PPRV-mediated unfolded protein response

**Figure 2:**
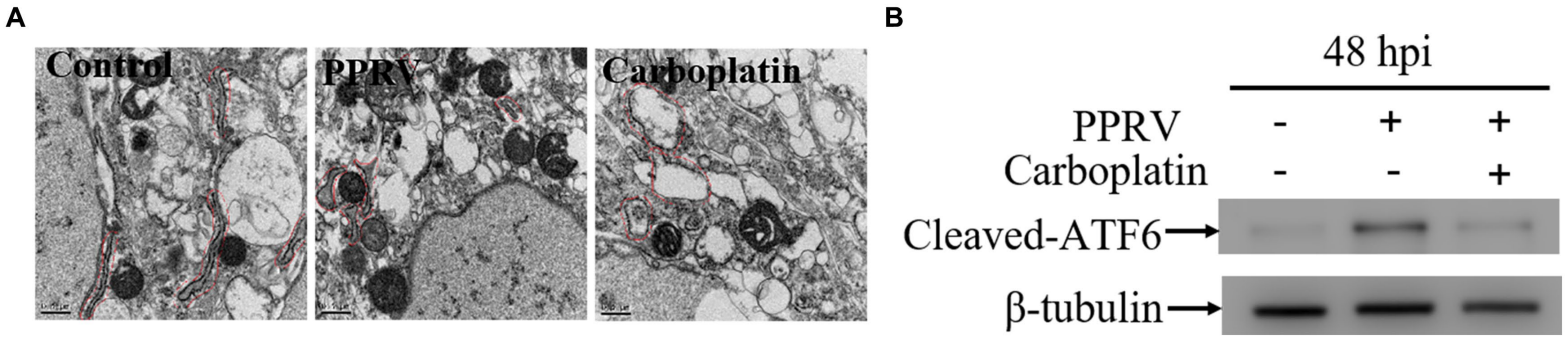
Carboplatin disturbs ER homeostasis and inhibits PPRV-induced ATF6 activation. **(A)** Transmission electron microscopy detection of morphological changes of ER. Vero cells pre-treated with Carboplatin at 100 μM or untreated were infected with PPRV at 1 MOI for 48 h and followed with treatment of TEM to observe the morphology of ER. **(B)** Western blotting detection of cleaved-ATF6 levels in PPRV infected (MOI = 1), Carboplatin-treated (100 μM) and untreated cells at 48 hpi. β-tubulin was used as a loading control.

Owing to the limited coding capacity of the viral genome, viruses co-opt host nuclear proteins for replication (48). As a protein synthesis factor, the multifunctional organelle endoplasmic reticulum (ER) is associated with several pathways involved in cellular homeostasis and survival (49). Therefore, we examined the effect of Carboplatin on ER homeostasis. Transmission electron microscopy assay results showed that the morphological swelling and dilation of ER in Carboplatin-treated PPRV-infected Vero cells was more severe than that observed in untreated PPRV-infected cells (Figure 2A), indicating that Carboplatin could effectively enhance PPRV-mediated disturbance of ER homeostasis. The ER is the largest cellular membrane network and contains the quality control machinery for protein folding and maturation. When misfolded or mutant proteins accumulate, perturbation of ER homeostasis can cause ER stress (50), which subsequently activates the evolutionarily conserved unfolded protein response (UPR) signaling pathways to alleviate the stress so that the cell can survive. Three transmembrane ER stress sensor proteins, PKR-like ER kinase (PERK), activating transcription factor 6 (ATF6), and inositol-requiring enzyme 1 (IRE1), control three arms of the UPR signaling pathway to resolve stress and maintain ER homeostasis (proteostasis) (51). A previous study demonstrated that PPRV infection can selectively activate the ATF6 branch of the UPR in Vero cells (45). To understand the possible molecular mechanisms involved in the antiviral activity of Carboplatin, we analyzed the expression level of activated ATF6, which is involved in cellular signal transduction of the UPR signaling pathway. Western blotting results revealed that the cleaved-ATF6 level in PPRV-infected cells treated with Carboplatin was significantly downregulated compared to that seen in PPRV-infected untreated cells (Figure 2B), which may partially explain the severity of the ER morphological changes in Carboplatin-treated PPRV-infected cells.

### Down-regulation of STING contributes to the antiviral effect of Carboplatin

**Figure 3:**
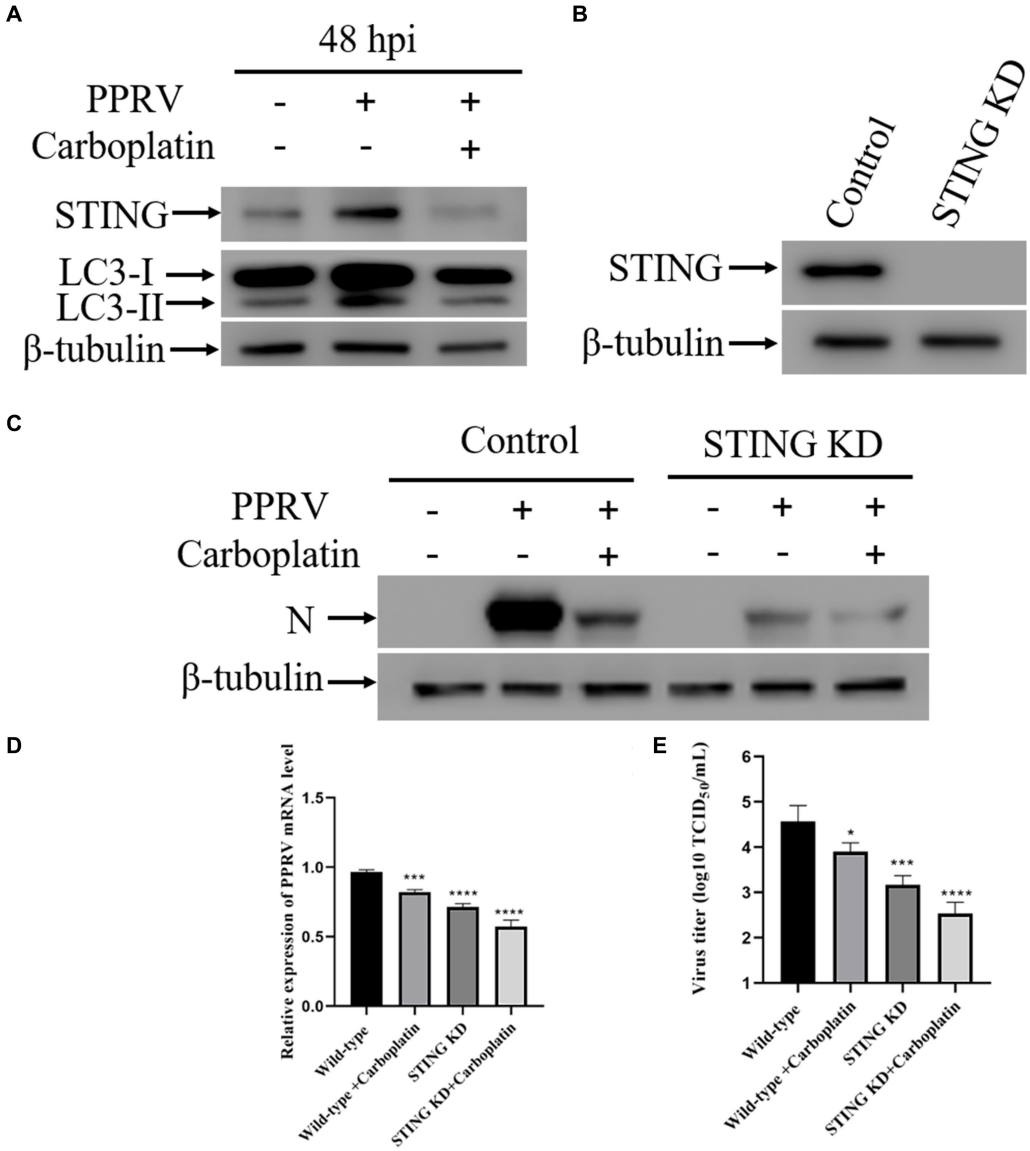
Down-regulation of STING contributes to the antiviral effect of Carboplatin. **(A)** Western blotting analysis of STING and LC3 in PPRV infected (MOI = 1), Carboplatin-treated (100 μM) and untreated cells at 48 hpi. β-tubulin was used as a loading control. **(B)** STING silencing efficiency were verified by Western boltting. β-tubulin was used as a loading control. **(C)** Western blotting analysis of PPRV N protein in PPRV-infected, Carboplatin-treated (100 μM) and untreated wild-type and STING KD cells (MOI = 1, 48 hpi). **(D)** PPRV mRNA levels in PPRV-infected, Carboplatin-treated (100 μM) and untreated wild-type and STING KD cells (MOI = 1, 48 hpi) were measured by qPCR. The data show the mean ± SD; *n* = 3; ***p < 0.001; ****p < 0.0001. **(E)** Wild-type and STING KD cells treated/untreated with Carboplatin (100 μM) were infected with PPRV (MOI = 1), and virus titers were measured by TCID_50_ (48 hpi). The data show the mean ± SD; *n* = 3; *p < 0.005; ***p < 0.001; ****p < 0.0001.

Autophagy is an evolutionarily conserved intracellular degradation process essential for the maintenance of cellular homeostasis through catabolic lysis of otherwise detrimental cytosolic components (43, 49, 52). UPR and autophagy are two different cellular programs that either work independently or coordinate to maintain cellular homeostasis in response to a diverse range of stresses. A growing number of studies have demonstrated that ER stress can initiates autophagy (53–55). Autophagy is associated with UPR by restricting protein production or removing misfolded proteins (49). Considering that the activation of ATF6 was inhibited by Carboplatin during PPRV infection, we reasoned that Carboplatin is involved in PPRV-induced autophagy. To test this hypothesis, Vero cells were treated with 100 μM Carboplatin for 24 h before infection with PPRV, and western blotting was performed to determine the conversion of LC3-I to LC3-II, which is currently regarded as an accurate indicator of autophagic activity (56). As shown in Figure 3A, compared with untreated infected cells, the band intensity of LC3-II in Carboplatin-treated, PPRV-infected Vero cells was dramatically decreased at 48 hpi, indicating that Carboplatin could inhibit PPRV-mediated autophagy induction in Vero cells. Our previous studies demonstrated that PPRV induces autophagy to facilitate viral replication by upregulating STING (45). To further elucidate the molecular mechanisms underlying the antiviral effects of Carboplatin, STING expression levels were assessed by western blotting. The results showed that the amount of STING protein was significantly reduced in PPRV-infected, Carboplatin-treated cells than in untreated PPRV-infected cells (Figure 3A), implying that Carboplatin might induce an antiviral response by suppressing STING expression. To confirm the role of STING in Carboplatin-induced antiviral activity, we generated STING stable knockdown Vero cells (Figure 3B) and analyzed viral replication levels. Western blotting results showed that the decrease in PPRV structural protein N was significantly enhanced by treatment of STING knockdown cells with Carboplatin at a concentration of 100 μM (Figure 3C). Moreover, Carboplatin treatment remarkably reduced PPRV mRNA levels in STING knockdown cells, as determined by qRT-PCR (Figure 3D). Meanwhile, Carboplatin treatment of infected STING knockdown cells resulted in the strongest reduction in viral titers in TCID_50_ analysis compared to Carboplatin-treated, PPRV-infected Vero, and PPRV-infected STING knockdown cells (Figure 3E). Together, these data indicate that the antiviral activity of Carboplatin is due to inhibition of STING upregulation induced by PPRV infection.

### Carboplatin inhibits PPRV-mediated autophagy via downregulating STING

**Figure 4:**
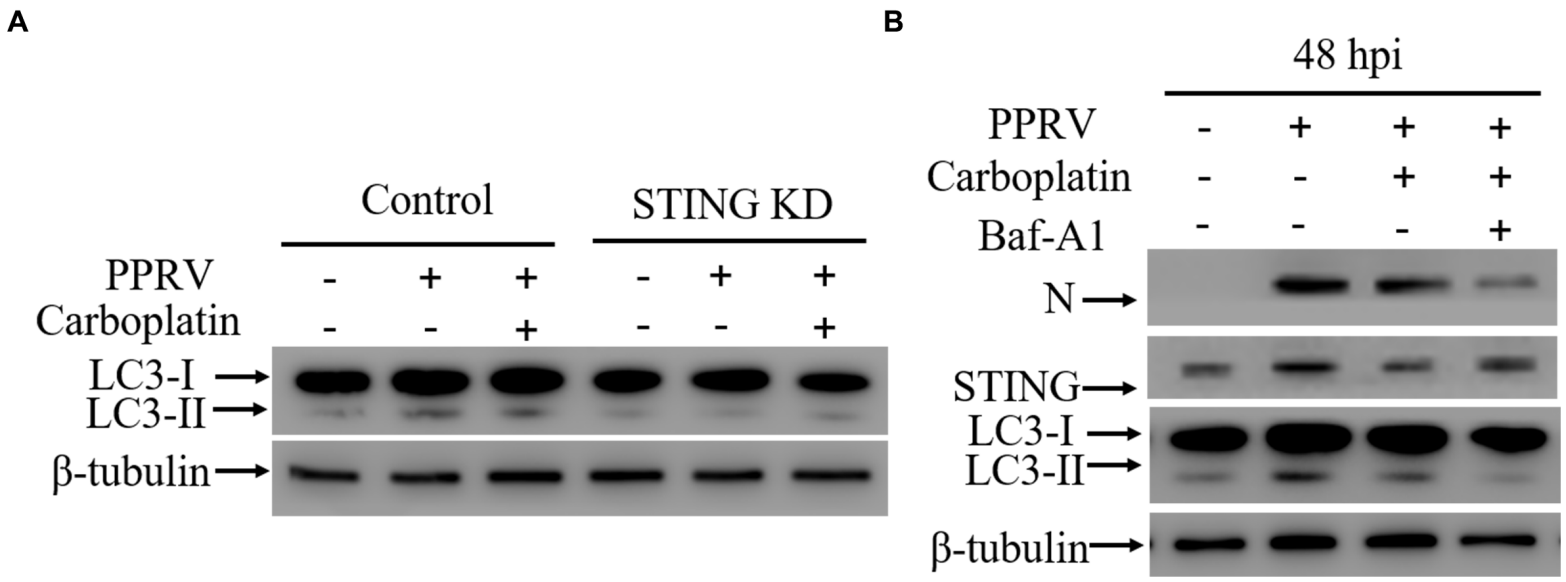
STING is essential for autophagy induction. **(A)** Western blotting analysis of LC3 in wild-type and STING KD cells treated/untreated with Carboplatin (100 μM) and infected with PPRV (MOI = 1, 48 hpi). β-tubulin was used as a loading control. **(B)** Western blotting detection of N, STING and LC3 in Vero cells infected with PPRV (48 hpi). Vero cells were pre-treated with or without Baf-A1 for 24 h before being treated with or without Carboplatin and infected with PPRV (MOI = 1). β-tubulin was used as a loading control.

Formally, as PPRV hijacks cellular autophagy for viral replication (8, 43–46), lower levels of viral loads could be due to inhibition of cellular autophagy flux or key autophagy-related molecules. Given that Carboplatin inhibits the PPRV-mediated upregulation of STING, and accumulating evidence has confirmed the indispensable role of STING in autophagy induction triggered by different cues (45, 57–59), we speculated that Carboplatin may exert antiviral activity by blocking PPRV-induced autophagy by inhibiting STING upregulation. To better understand the molecular mechanism involved in PPRV-mediated autophagy inhibition by Carboplatin, we established STING knockdown cells using shRNA and examined whether STING was also involved in the inhibition of autophagy by Carboplatin. As expected, western blotting analysis showed that knockdown of STING suppressed autophagy in PPRV-infected Vero cells, and enhanced the inhibitory effect of Carboplatin on autophagy. PPRV infection was unable to induce autophagy in Carboplatin-treated STING knockdown cells in comparison with Carboplatin-treated wild-type control cells (Figure 4A), indicating that Carboplatin inhibits PPRV-induced autophagy by downregulating STING. Moreover, inhibition of PPRV-induced autophagy with bafilomycin A1 (Baf-A1), an autophagy inhibitor, resulted in a greater reduction in the amount of N protein when compared to Vero cells treated with Carboplatin alone (Figure 4B). Taken together, these findings suggest that the antiviral activity of Carboplatin is due to the potent inhibition of STING upregulation and its ability to induce autophagy during PPRV infection.

### Inhibition of autophagy is responsible for the antiviral activity of Carboplatin

**Figure 5:**
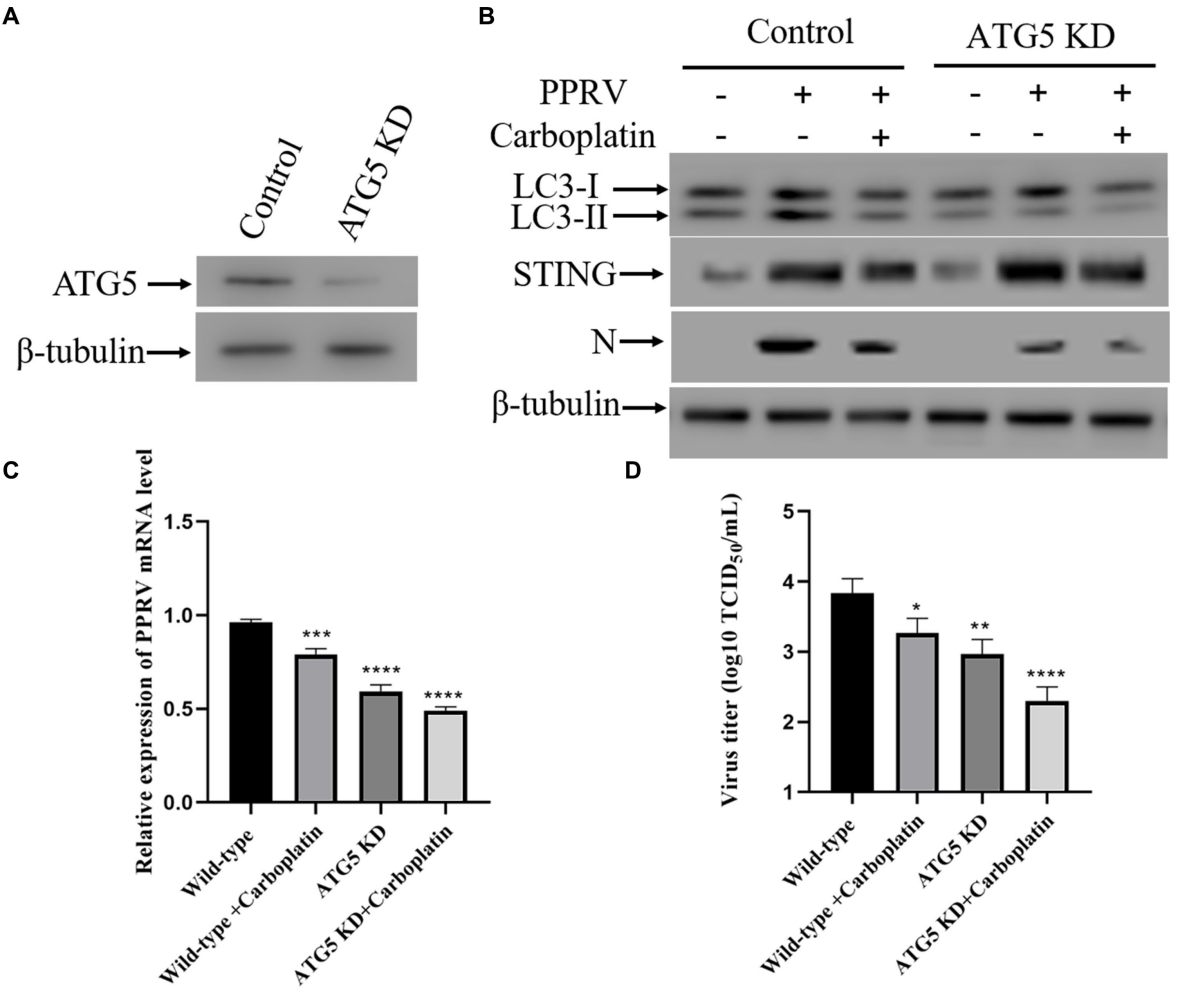
Inhibition of autophagy is responsible for the reduced production of PPRV. **(A)** ATG5 silencing efficiency were verified by Western boltting. β-tubulin was used as a loading control. **(B)** Western blotting analysis of PPRV N protein, LC3 and STING in PPRV-infected, Carboplatin-treated (100 μM) and untreated wild-type and ATG5 KD cells (MOI = 1, 48 hpi). **(C)** PPRV mRNA levels in PPRV-infected, Carboplatin-treated (100 μM) and untreated wild-type and ATG5 KD cells (MOI = 1, 48 hpi) were measured by qPCR. The data show the mean ± SD; *n* = 3; ***p < 0.001; ****p < 0.0001. **(D)** Wild-type and ATG5 KD cells treated/untreated with Carboplatin (100 μM) were infected with PPRV (MOI = 1), and virus titers were measured by TCID_50_ (48 hpi). The data show the mean ± SD; *n* = 3; *p < 0.005; **p < 0.01; ****p < 0.0001.

At the molecular level, autophagy is a fine-orchestrated process that involves numerous proteins, including those encoded by autophagy-related genes (ATG) (60). Upon autophagy activation, ATGs are recruited to subdomains close to the ER to play essential roles (61). The core autophagy machinery protein ATG5 is critical for autophagosome formation and is responsible for phagophore elongation. ATG5 knockdown blocks autophagy (62). To further confirm the role of autophagy in the antiviral activity of Carboplatin, inhibition of autophagy was achieved by transducing shRNA targeting ATG5 into Vero cells, and the protein level of ATG5 was assessed via western blotting (Figure 5A). As shown in Figure 5B, ATG5 knockdown suppressed autophagy and PPRV N protein expression in Vero cells. Furthermore, the absence of ATG5 promoted the inhibitory effect of Carboplatin and led to the highest reduction in N protein levels compared to Carboplatin-treated, PPRV-infected Vero cells, and PPRV-infected ATG5 knockdown cells. In accordance with this, treatment of ATG5 knockdown cells with Carboplatin resulted in the lowest level of the autophagy marker LC3-II. However, inhibition of PPRV-induced STING upregulation by Carboplatin was not affected in ATG5-deficient cells (Figure 5B). Moreover, compared with those in the virus-only controls, the mRNA levels of PPRV were significantly decreased in Carboplatin-treated, PPRV-infected cells as determined by qRT-PCR, and the strongest decrease was observed in Carboplatin-treated, PPRV-infected ATG5 knockdown cells (Figure 5C). Similarly, treatment of ATG5 knockdown cells with Carboplatin resulted in the highest reduction in viral titers in the TCID_50_ assay (Figure 5D). In summary, these findings imply that the anti-PPRV activity of Carboplatin is attributable to its ability to inhibit PPRV-induced autophagy.

## Discussion

PPRV, a highly contagious and deadly virus in sheep and goats, is considered a great threat to small ruminants worldwide and has already caused huge economic losses in endemic regions worldwide (2, 9). Currently, the only available option for the control of PPRV infection and spreading relies on vaccination. Live attenuated vaccines have already been widely used to control PPR and are recognized as key tools in the global PPR eradication program (63, 64). However, like all paramyxoviruses, PPRV is heat sensitive and therefore an effective cold chain is needed to deliver the live attenuated vaccine in areas with a hot climate, which results in significantly increased costs of the vaccine (21). These observations highlight the urgent need for the development of antiviral agents that can effectively prevent PPRV infection. However, there are no data regarding PPRV-targeting antiviral drugs.

Carboplatin, a second clinically important platinum analog, has come into common clinical use and has become the mainstay treatment for many tumors (29, 65). Notably, Carboplatin has been proven to be the safest platinum drug for use in pregnancy (31, 32). Although it was developed for humans, studies have demonstrated that Carboplatin is also an efficient anticancer agent of dogs and cats, as Carboplatin therapy for canine appendicular osteosarcoma and feline oral and cutaneous squamous cell carcinomas has shown promising clinical efficiency (28, 35, 36). It is well known that chemotherapy induces antitumor immune response (66). Carboplatin can promote antitumor immune response by reducing immunoinhibitory cells (37, 67–69). Moreover, study has shown that the platinum-based chemotherapeutic agent can modulate viral replication in chronic hepatitis B patients undergoing chemotherapy (38). However, the effect of Carboplatin on immunosuppressive morbilliviruses replication remains unknown. In the present study, we analyzed the influence of Carboplatin in morbilliviruses infection and found for the first time that Carboplatin inhibits PPRV replication in Vero cells.

The ER is a dynamic organelle responsible for protein biosynthesis in eukaryotic cells (70). Diverse cellular stresses, including microbial infection and protein folding defects, can disrupt ER homeostasis and ultimately result in ER stress (71). During productive infection, viruses produce a large number of viral proteins that accumulate in the ER lumen and finally give rise to ER stress (72, 73). To buffer ER stress and orchestrate the recovery of ER function, the UPR is activated by inhibiting global translation (74). The UPR consists of three pathways: PERK, IRE1, and ATF6 (75, 76). Mounting evidence has illustrated that various viruses regulate the UPR to promote their replication (77–79), such as porcine epidemic diarrhea virus (PEDV) (55), dengue virus (DENV) (53), Japanese encephalitis virus (JEV) (80) and HBV (54). Another member of the *Morbillivirus*, CDV, dramatically expresses H and F proteins and accumulates in the ER, triggering UPR (81). We previously demonstrated that PPRV infection induces ER stress and selectively activates the ATF6 pathway of the UPR to promote viral infection in Vero cells (43, 45). Similarly, the ATF6 pathway is activated during enterovirus A71 (EV-A71) infection, with proteolytic cleavage of ATF6 (48, 82). Our results showed that as a functional consequence of reduced PPRV replication, inhibition of ATF6 activation and more serious morphological changes in the ER were observed in Carboplatin-treated Vero cells. Another study showed that it was the PERK/eIF2α pathway but not the ATF6 or IRE1 pathway that involved in activating ER stress-mediated autophagy to enhance PPRV replication in EECs (43), suggesting that the activation of ER stress-mediated UPR may be with cell-type specificity during PPRV infection.

Autophagy is regarded as an fundamental cellular response to fight microbial infection by degrading infectious pathogens sequestered within autophagosomes and plays a key role in the induction of both innate and adaptive immune response (52, 83, 84). Numerous viruses have evolved strategies to counteract the autophagic pathway to facilitate their own replication, such as MeV (43, 46, 85), DENV (86), foot-and-mouth disease virus (FMDV) (87), porcine circovirus type 2 (PCV-2) (88), coxsackievirus B3 (CVB3) (89), hepatitis C virus (HCV) (90), classical swine fever virus (CSFV) (91), porcine reproductive and respiratory syndrome virus (PRRSV) (92), avian reovirus (ARV) (93) and influenza A virus (IAV) (94). In the study by Chen et al., they found that the platinum-based chemotherapeutic agent Cisplatin promotes HBV replication via inducing autophagy in patients receiving chemotherapy (38). Here, we found that Carboplatin suppresses PPRV replication by inhibiting the autophagy pathway in Vero cells. Similarly, Yang et al. reported that treatment with autophagy inhibitors NH4Cl, chloroquine and wortmannin led to significantly decrease of structural protein N in PPRV-infected EECs (44). Zhang et al. found that inhibition of autophagy with small interfering RNA (siRNA) targeting ATG7 contributed to a significant reduction in the expression of PPRV N protein as well as the yield of progeny virions in Vero cells (46). Moreover, it has recently been shown that treatment of Vero cells with chloroquine and wortmannin resulted in a dramatically decrease of N protein and viral titer in CDV-infected cells (95). In G. Ferrara’s work, they observed a decrease of viral yield and viral proteins in permissive cells pretreated with autophagy inhibitors (bafilomycin, chloroquine and 3-methyladenine) during feline herpesvirus type 1 (FeHV-1) infection (96, 97). Pseudorabies virus (PRV) activates autophagy to elevate viral replication and inhibition of autophagy with 3-methyladenine (3-MA) restrained PRV replication in mouse neuro-2a cells (98).

STING, an evolutionarily conserved transmembrane protein localized in the ER membrane of immune and non-immune cells (99), is best known for its important signaling adaptor function in the activation of type I interferon responses to infection with DNA viruses (100). Apart from the classical role in mediating interferon and pro-inflammatory cytokines production, studies have revealed that autophagy induction is an evolutionarily conserved function of STING (58, 59, 101, 102). Moretti et al. found that during gram-positive bacterium *L. innocua* infection, STING is required to activate the PERK-mediate ER stress response and ultimately leads to reticulophagy (101). In our previous study, we have demonstrated that STING interacts with PERK to activate ER stress-mediated autophagy in response to FMDV infection (102). Unlike positive sense ssRNA virus (FMDV), PPRV (negative sense ssRNA virus) upregulates STING to activate ATF6-induced autophagy (45). Our results showed that Carboplatin blocks autophagy by inhibiting the PPRV-induced upregulation of STING and subsequent ATF6 activation, however, the precise mechanism by which Carboplatin inhibits STING-mediated autophagy requires further investigation.

In conclusion, we demonstrated for the first time that Carboplatin effectively inhibits PPRV replication *in vitro*. Evaluation of the mechanism of action of Carboplatin against PPRV revealed that the antiviral activity is due to the inhibition of autophagy by inhibiting the upregulation of STING induced by PPRV, highlighting that modulation of STING represents an attractive approach to counteract both DNA and RNA viruses. Since Carboplatin is a clinically approved drug for antitumor treatment, our data might provide a new therapeutic option for the cure and possibly also the prevention of viral diseases in humans.

## Data availability statement

The datasets presented in this study can be found in online repositories. The names of the repository/repositories and accession number(s) can be found at: https://www.jianguoyun.com/p/DdKdth4Qi4mvDBjom8IFIAA.

## Author contributions

RZ: Funding acquisition, Writing – original draft, Writing – review & editing. ZH: Project administration, Writing – review & editing. DW: Project administration, Writing – review & editing. RL: Project administration, Writing – review & editing. YL: Data curation, Writing – review & editing. ZZ: Methodology, Writing – review & editing.
