# Supplementary material for: Carboplatin restricts peste des petits ruminants virus replication by suppressing the STING-mediated autophagy
Source: Front Vet Sci. 2024 May 15;11:1383927. doi: 10.3389/fvets.2024.1383927 (PMC11133560; doi:10.3389/fvets.2024.1383927)
Supplement: Supplementary file 1 [file Presentation_1.PPTX]

## Slide 1
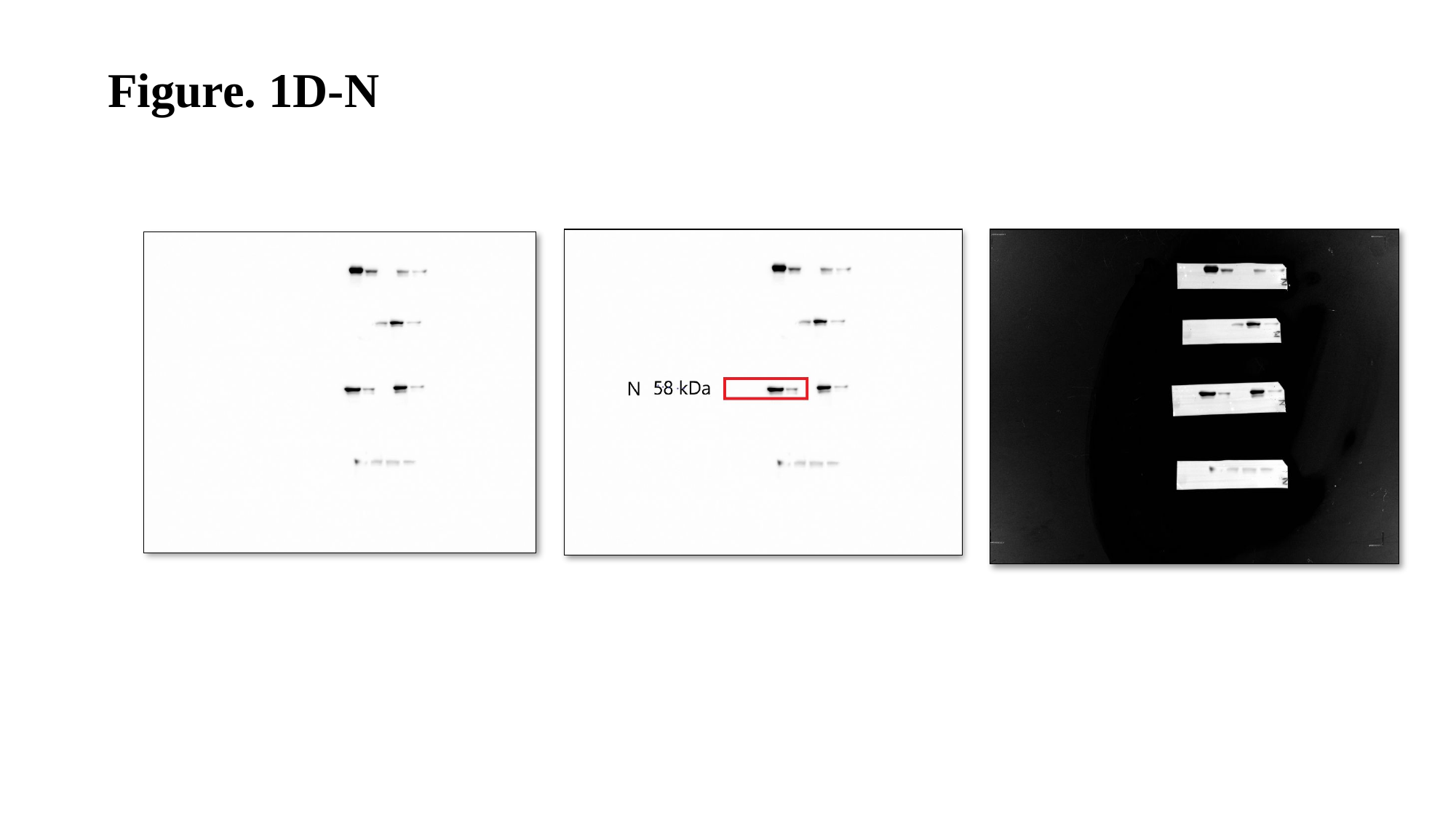

Figure. 1D-N

## Slide 2
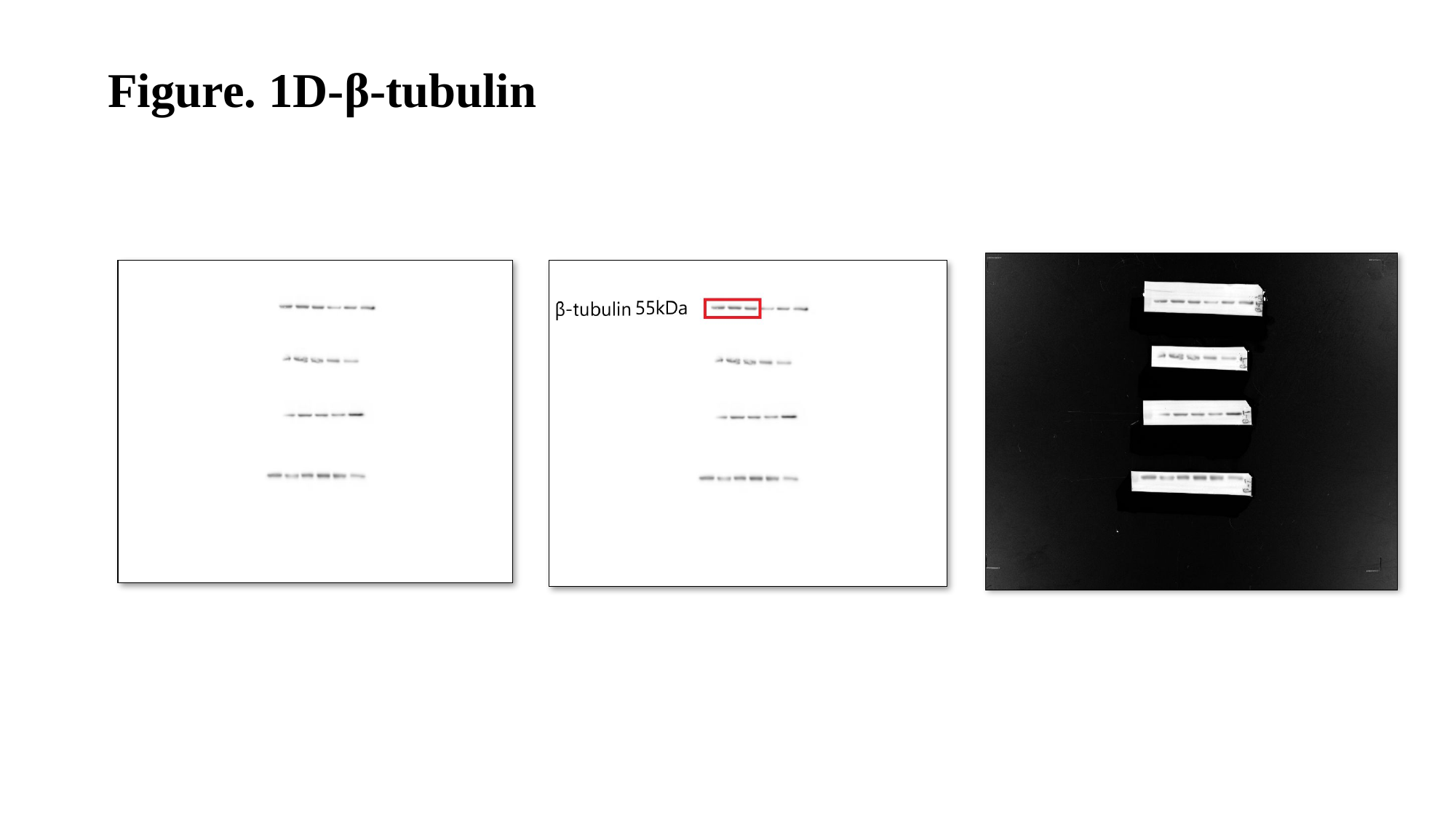

Figure. 1D-β-tubulin

## Slide 3
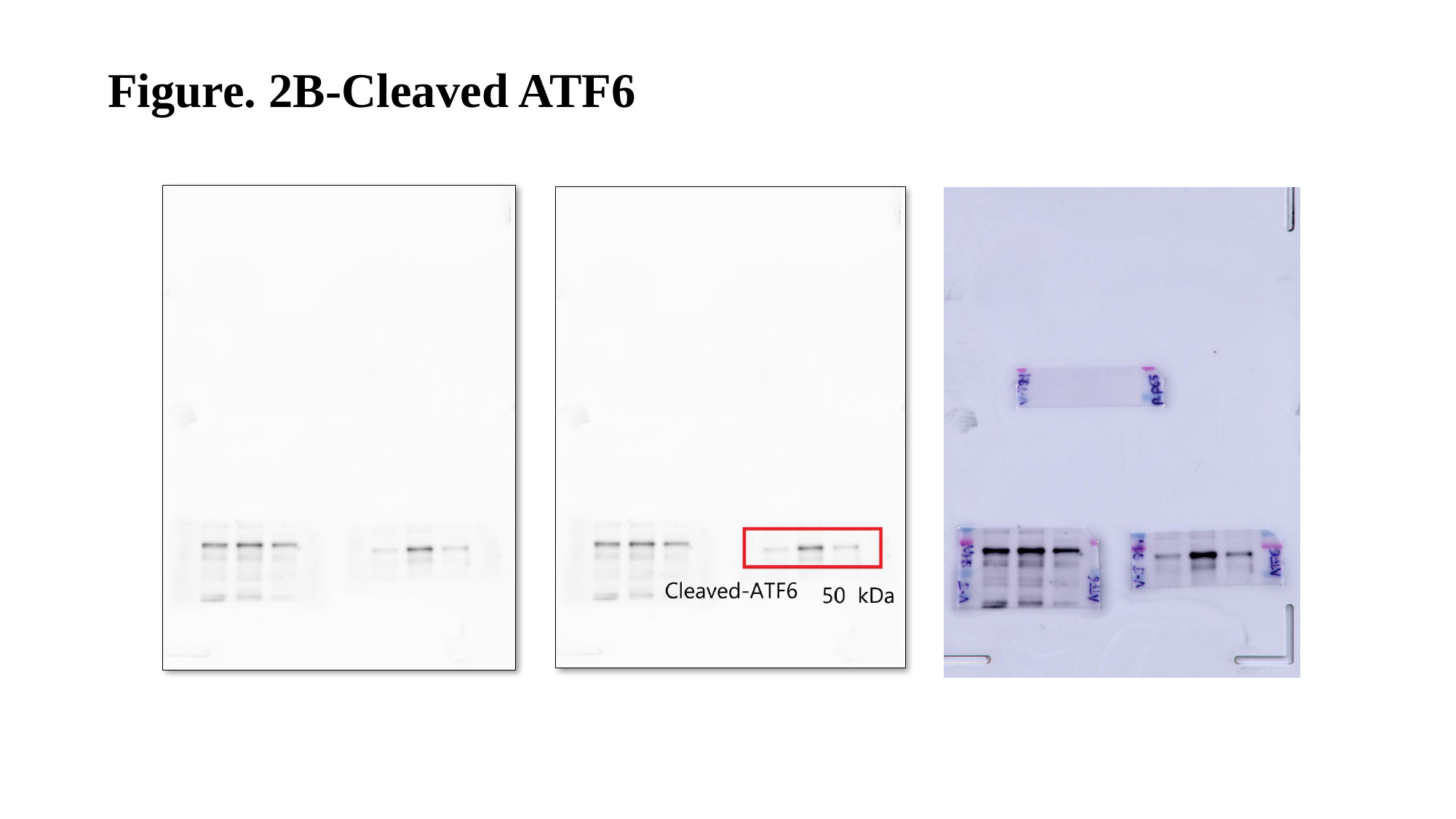

Figure. 2B-Cleaved ATF6

## Slide 4
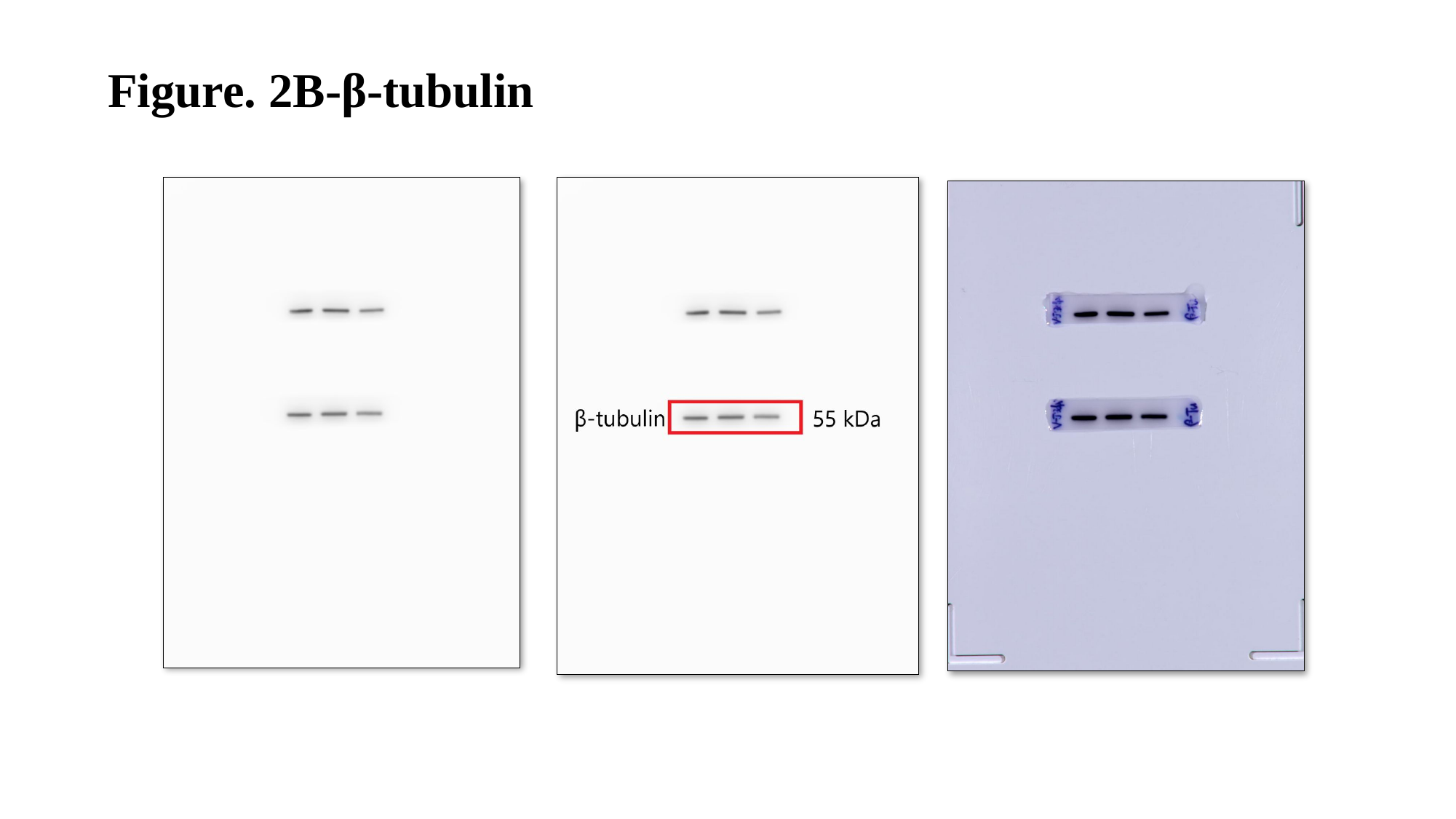

Figure. 2B-β-tubulin

## Slide 5
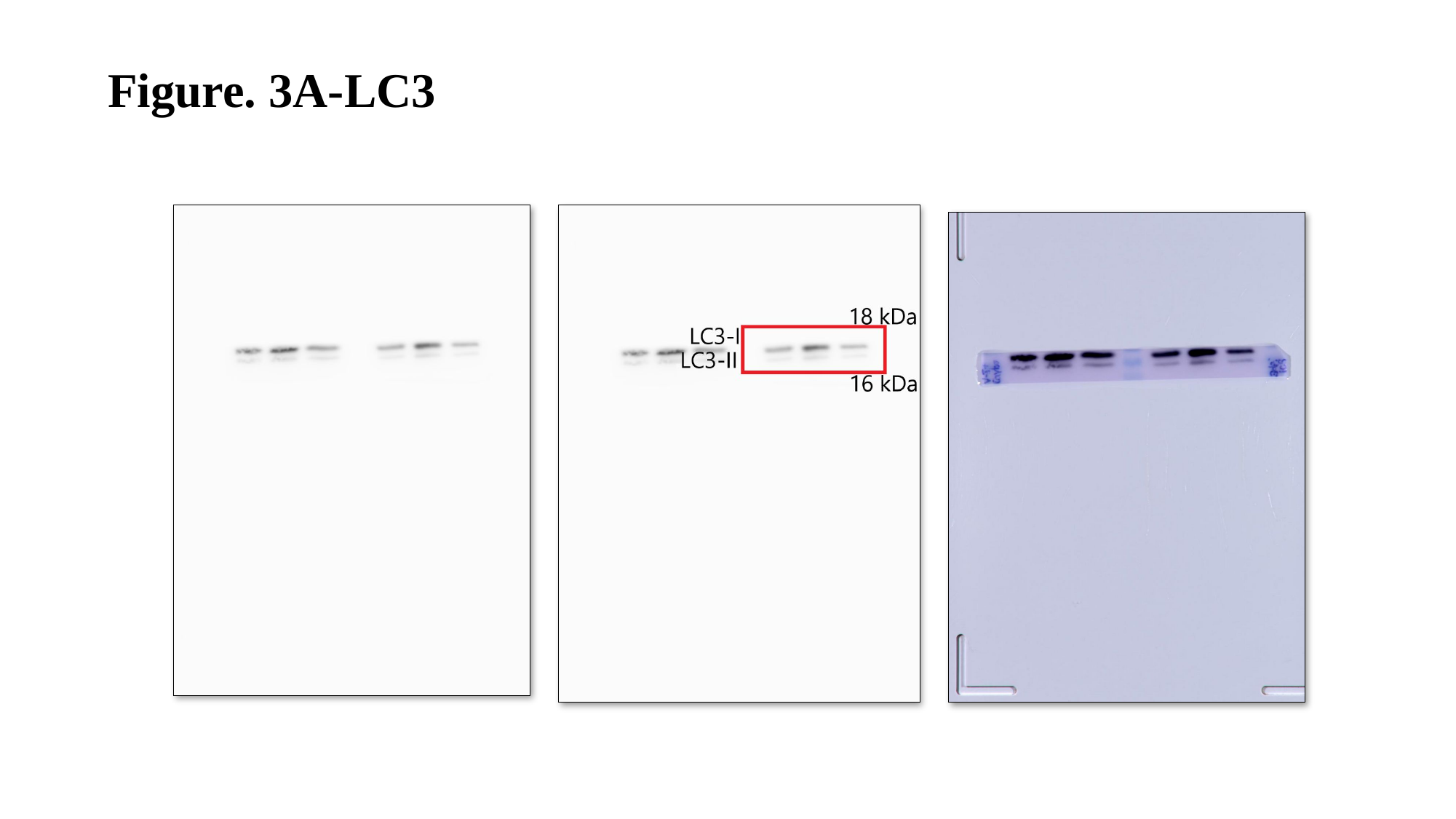

Figure. 3A-LC3

## Slide 6
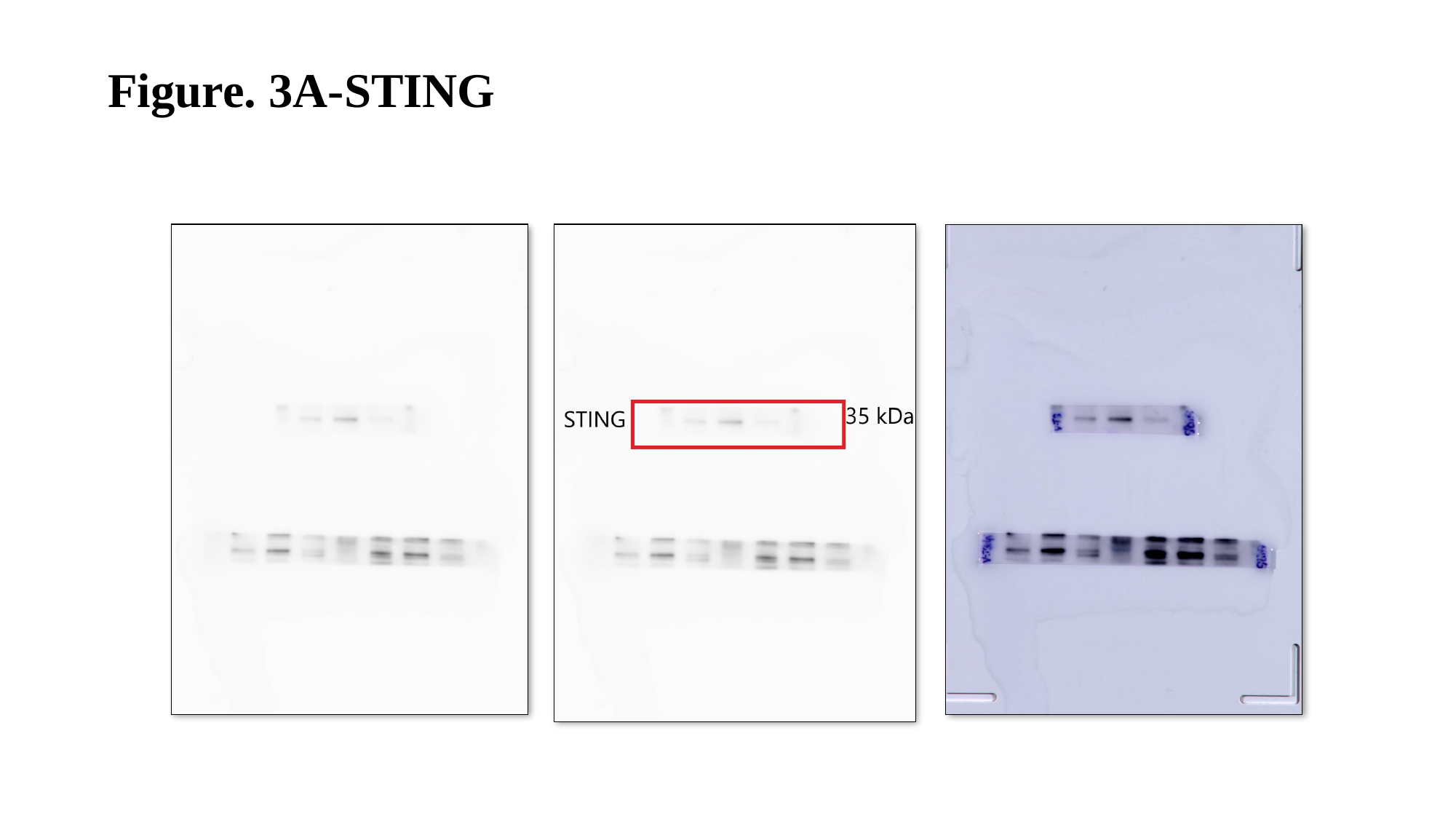

Figure. 3A-STING

## Slide 7
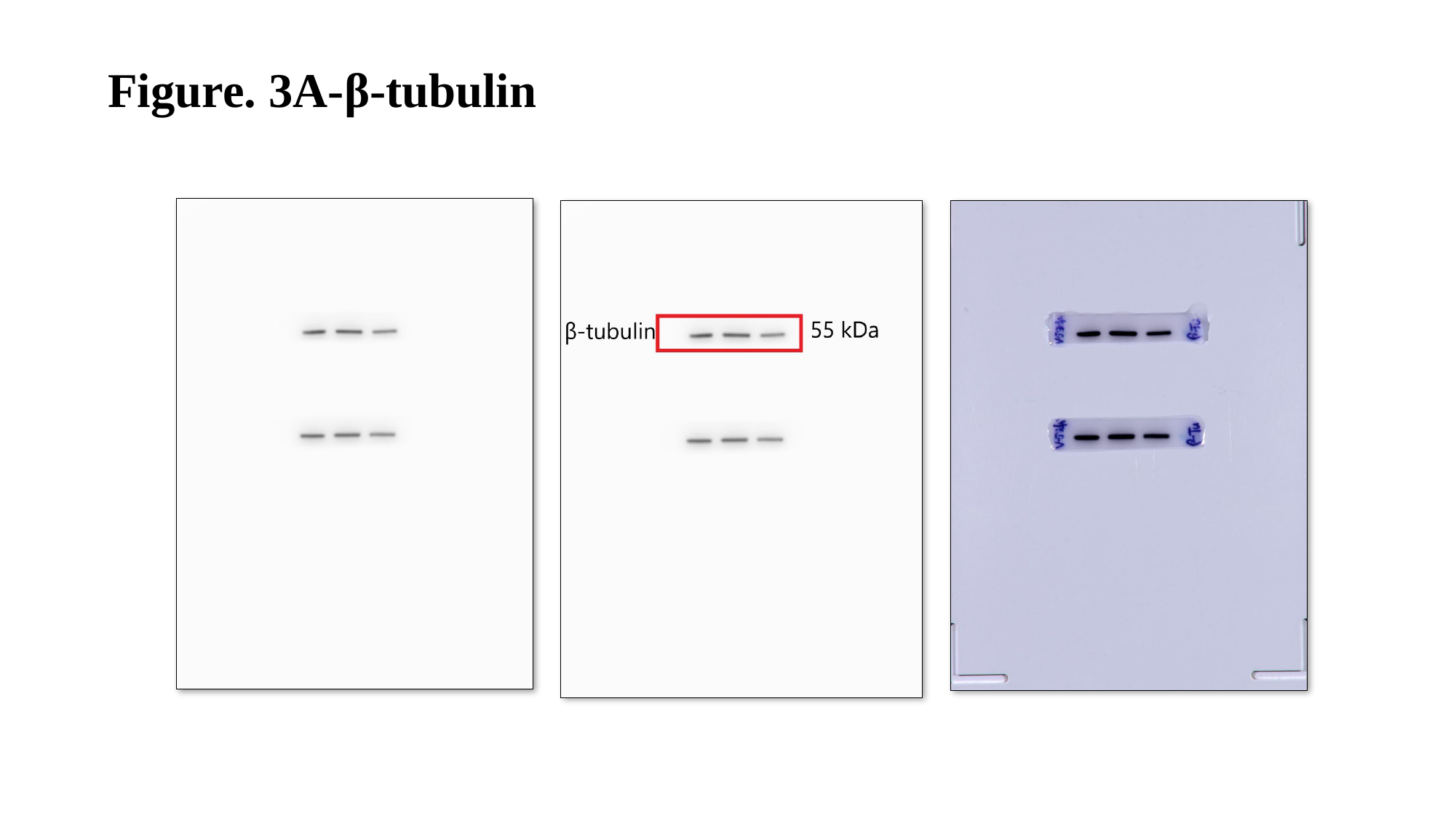

Figure. 3A-β-tubulin

## Slide 8
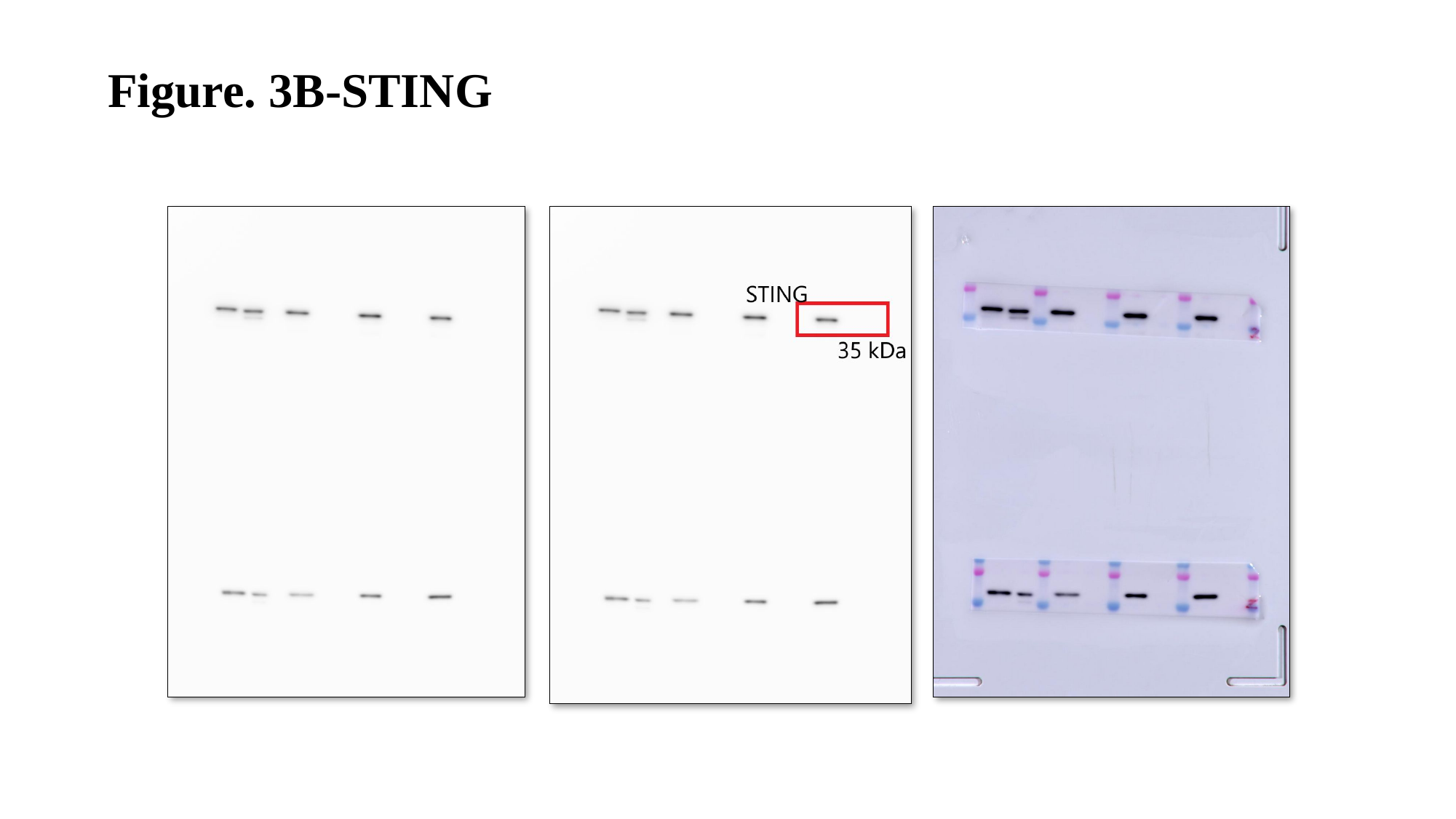

Figure. 3B-STING

## Slide 9
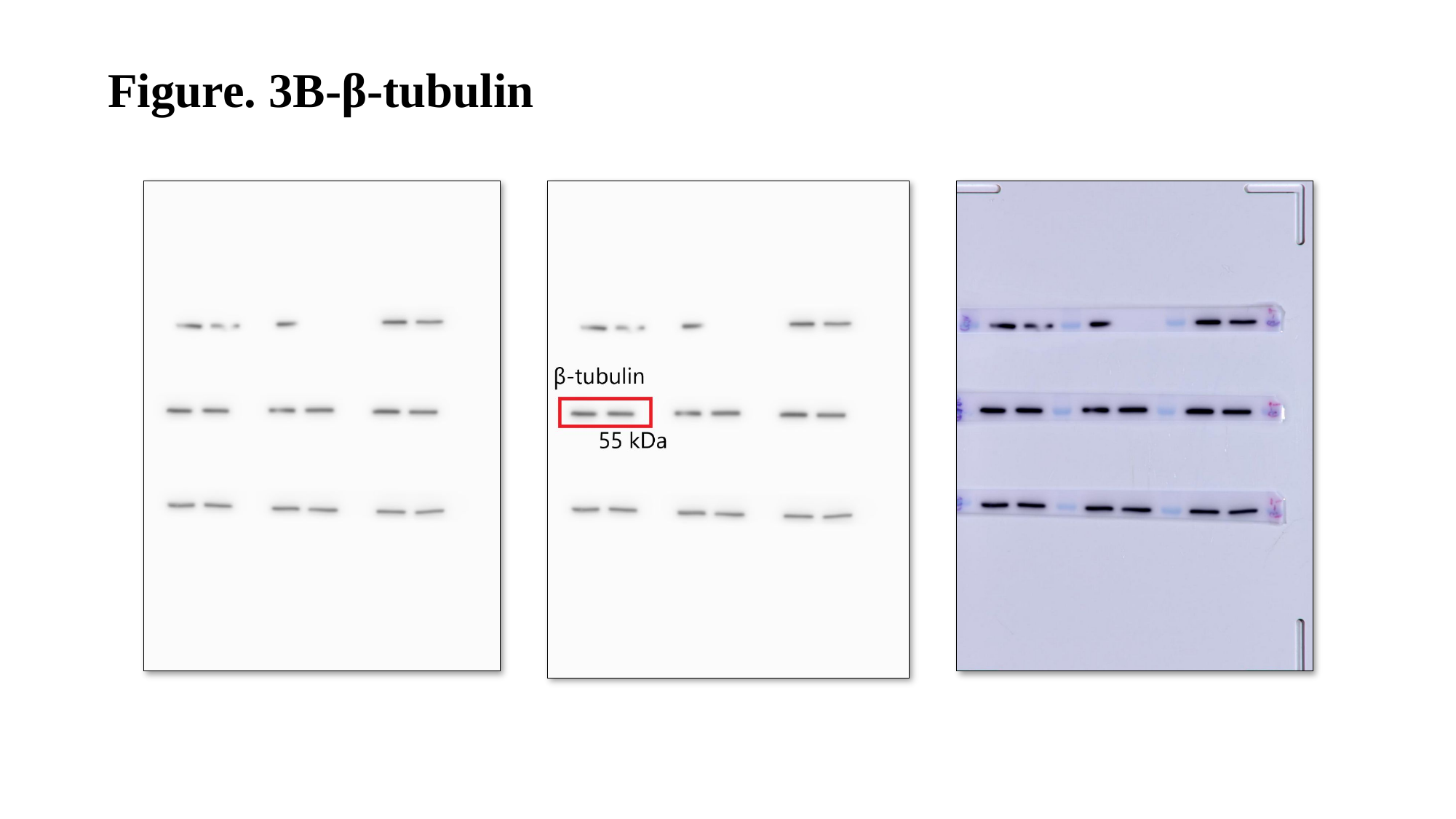

Figure. 3B-β-tubulin

## Slide 10
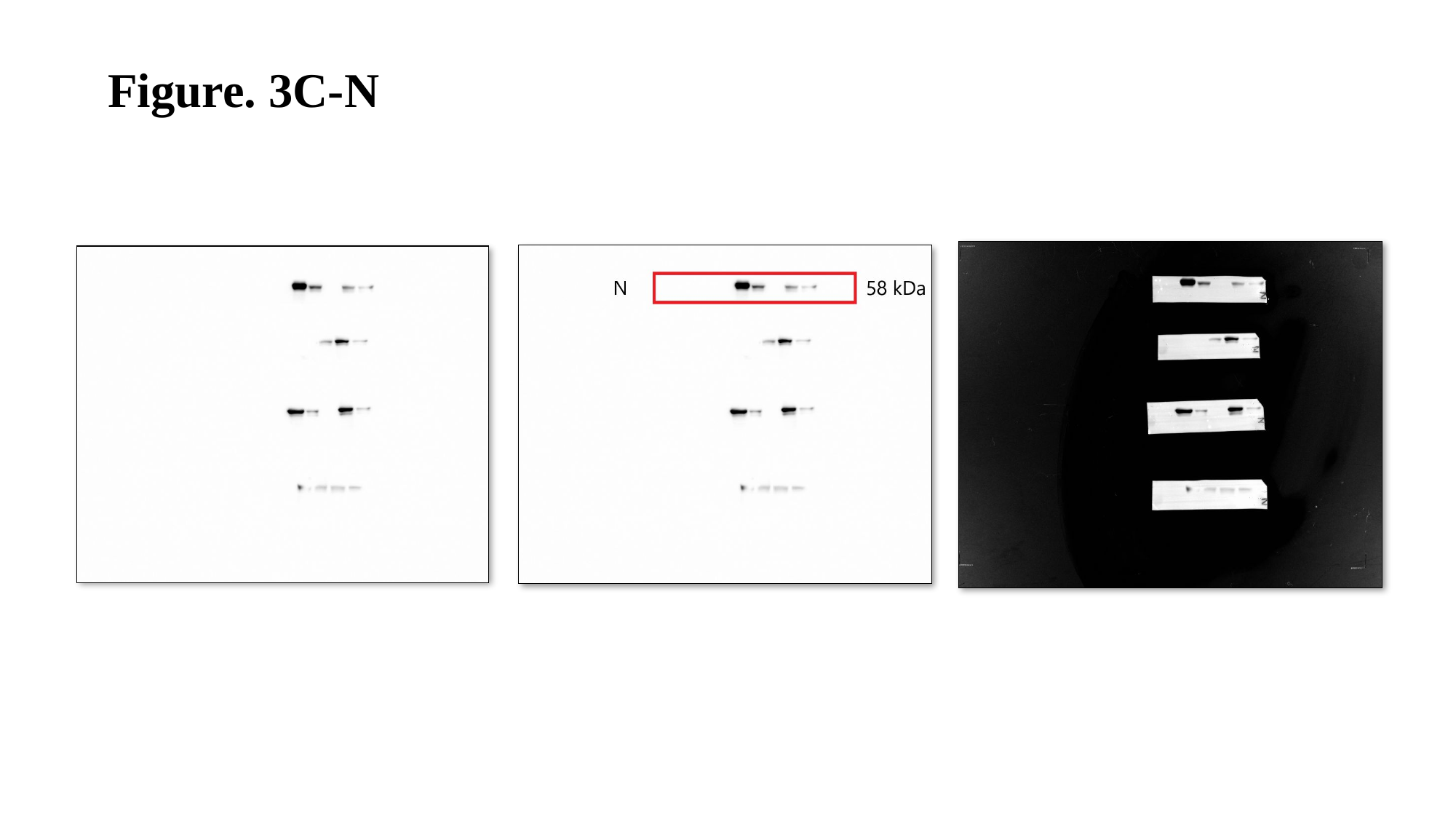

Figure. 3C-N

## Slide 11
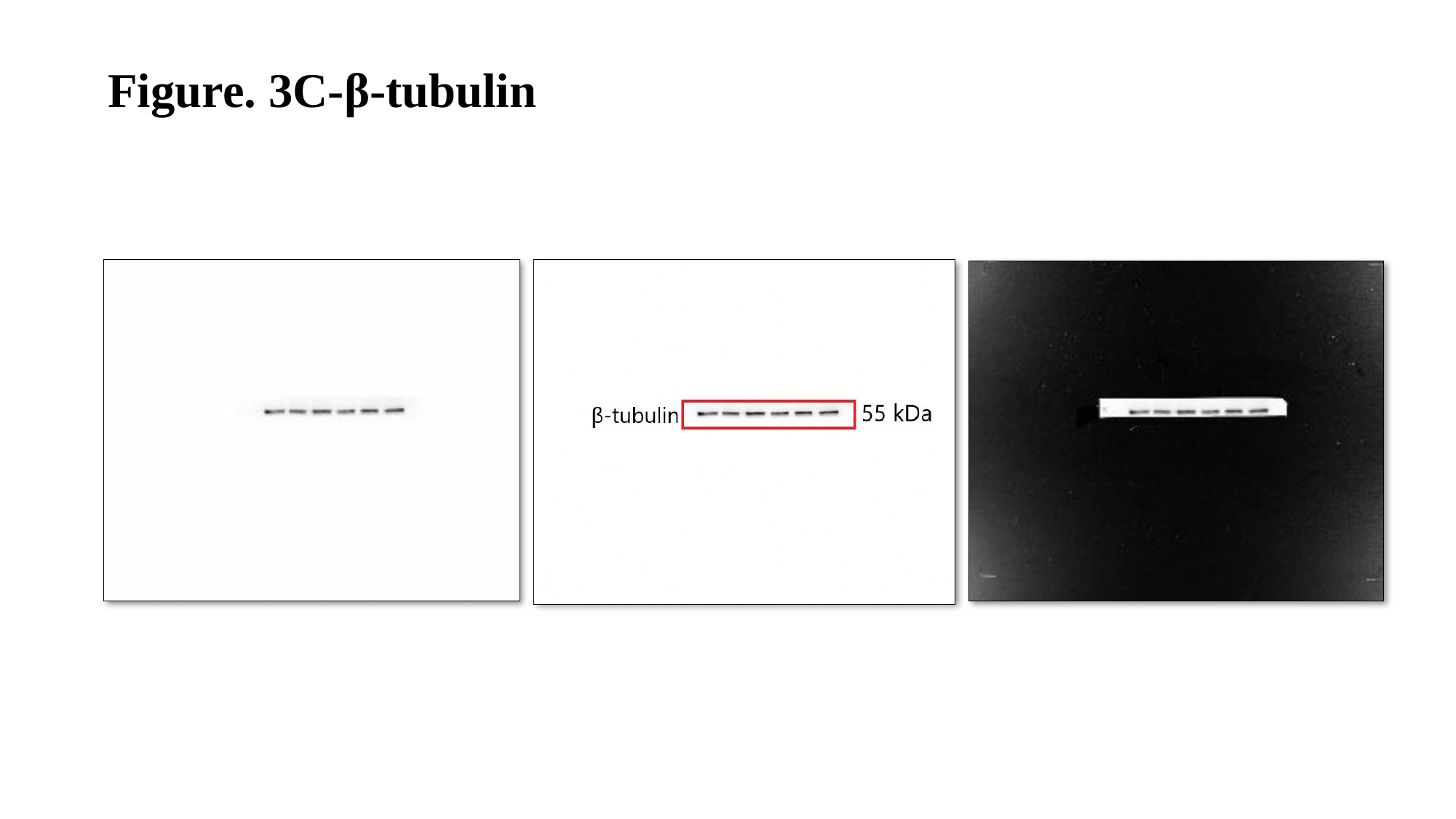

Figure. 3C-β-tubulin

## Slide 12
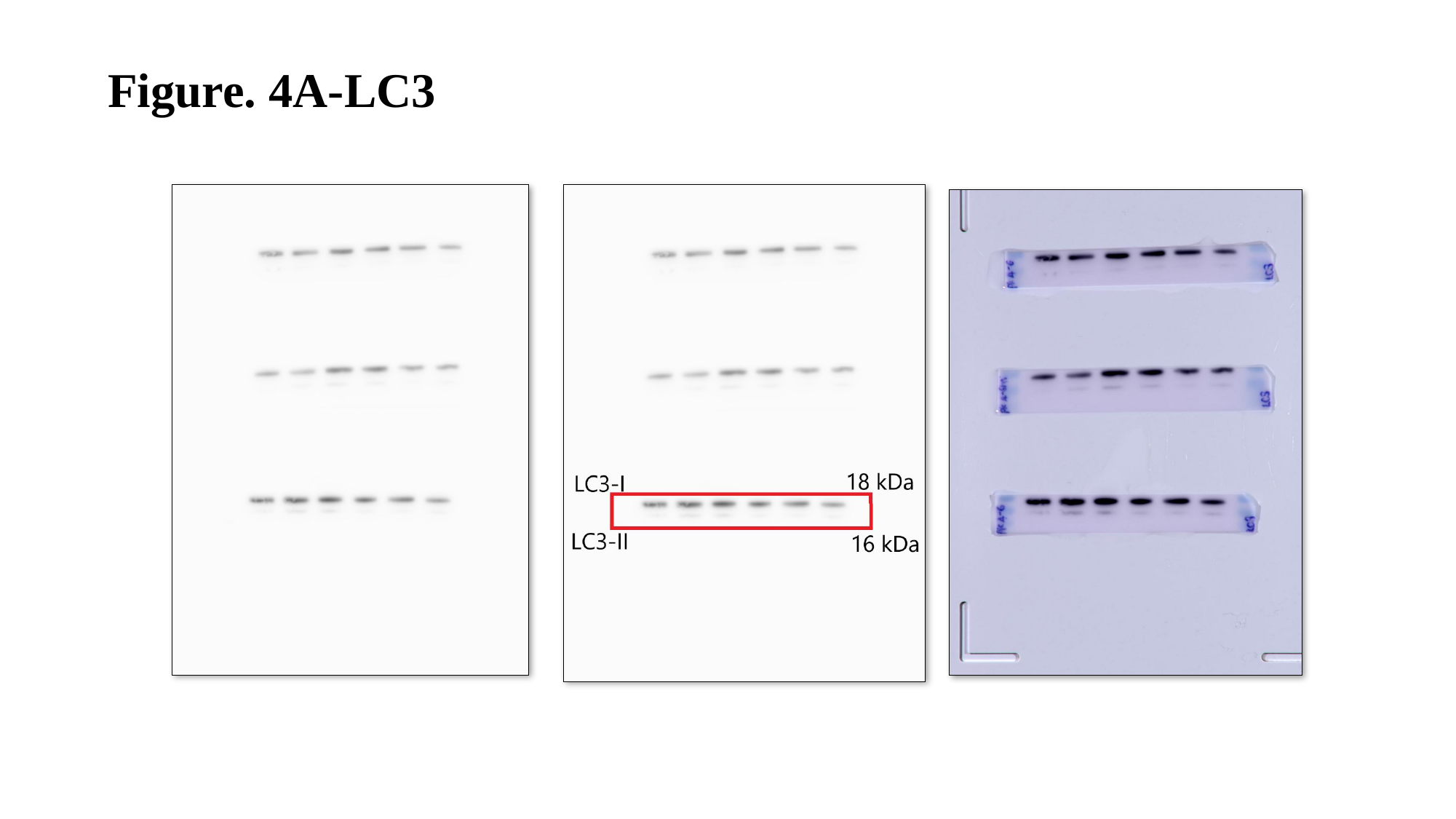

Figure. 4A-LC3

## Slide 13
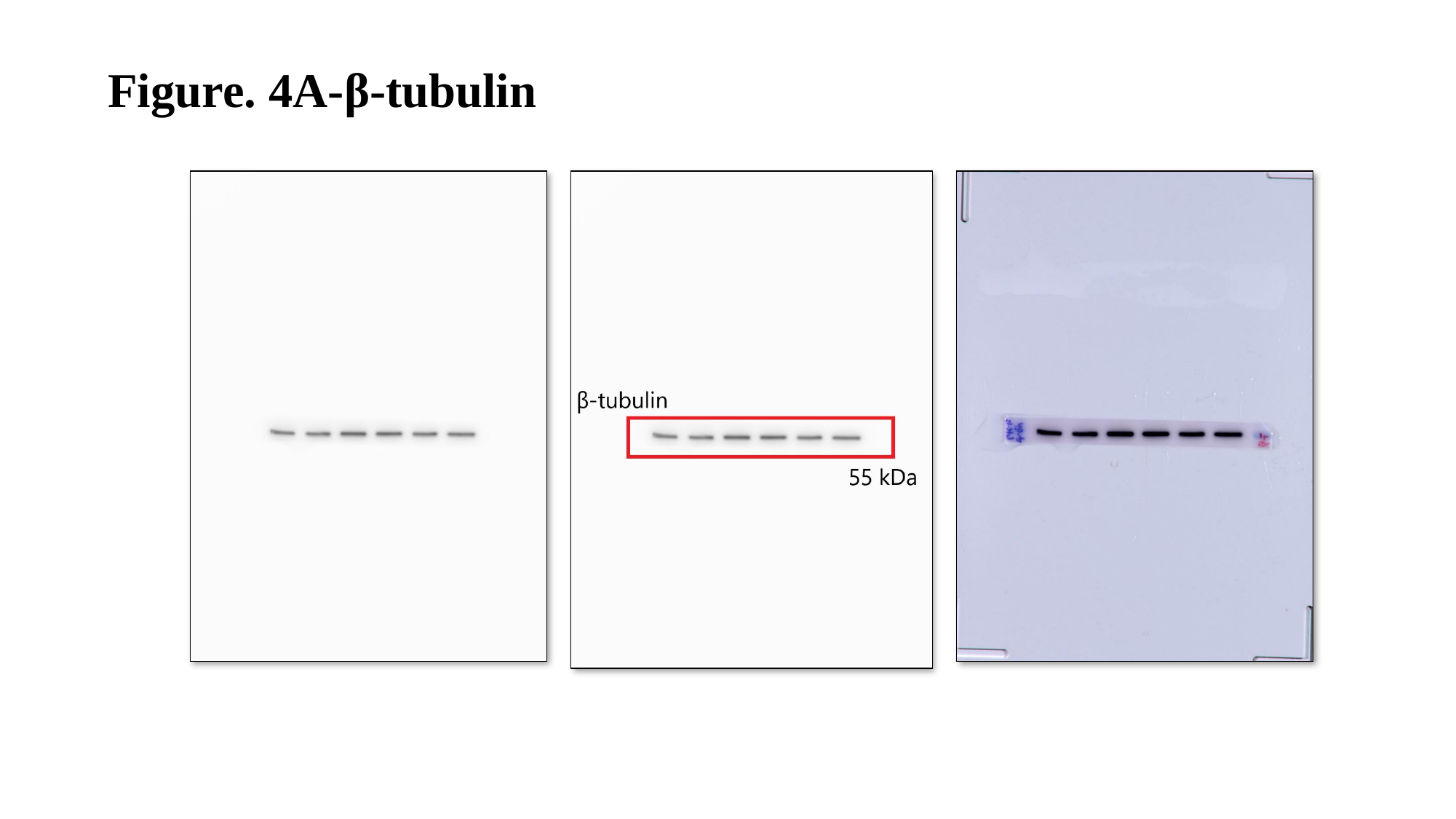

Figure. 4A-β-tubulin

## Slide 14
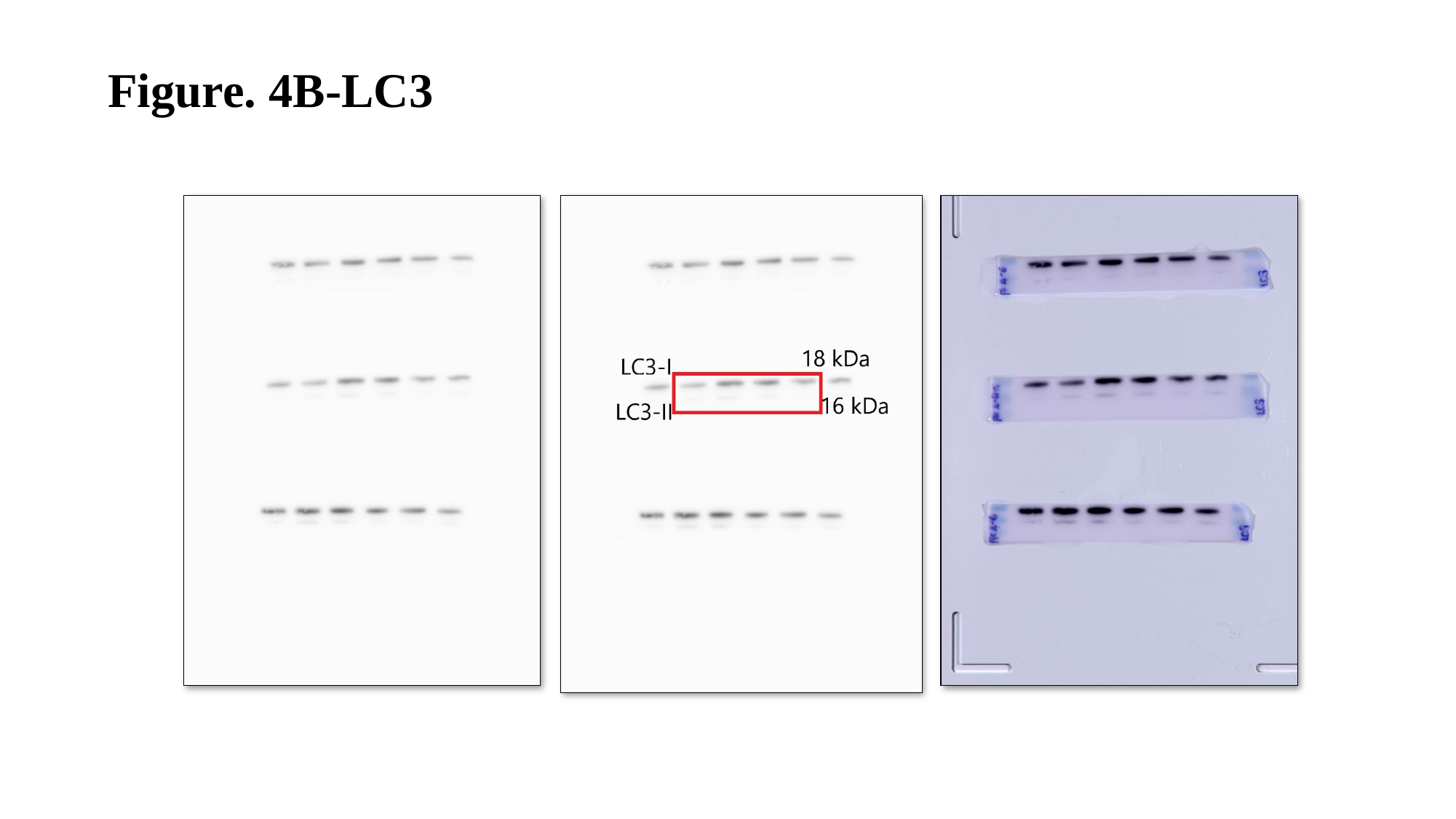

Figure. 4B-LC3

## Slide 15
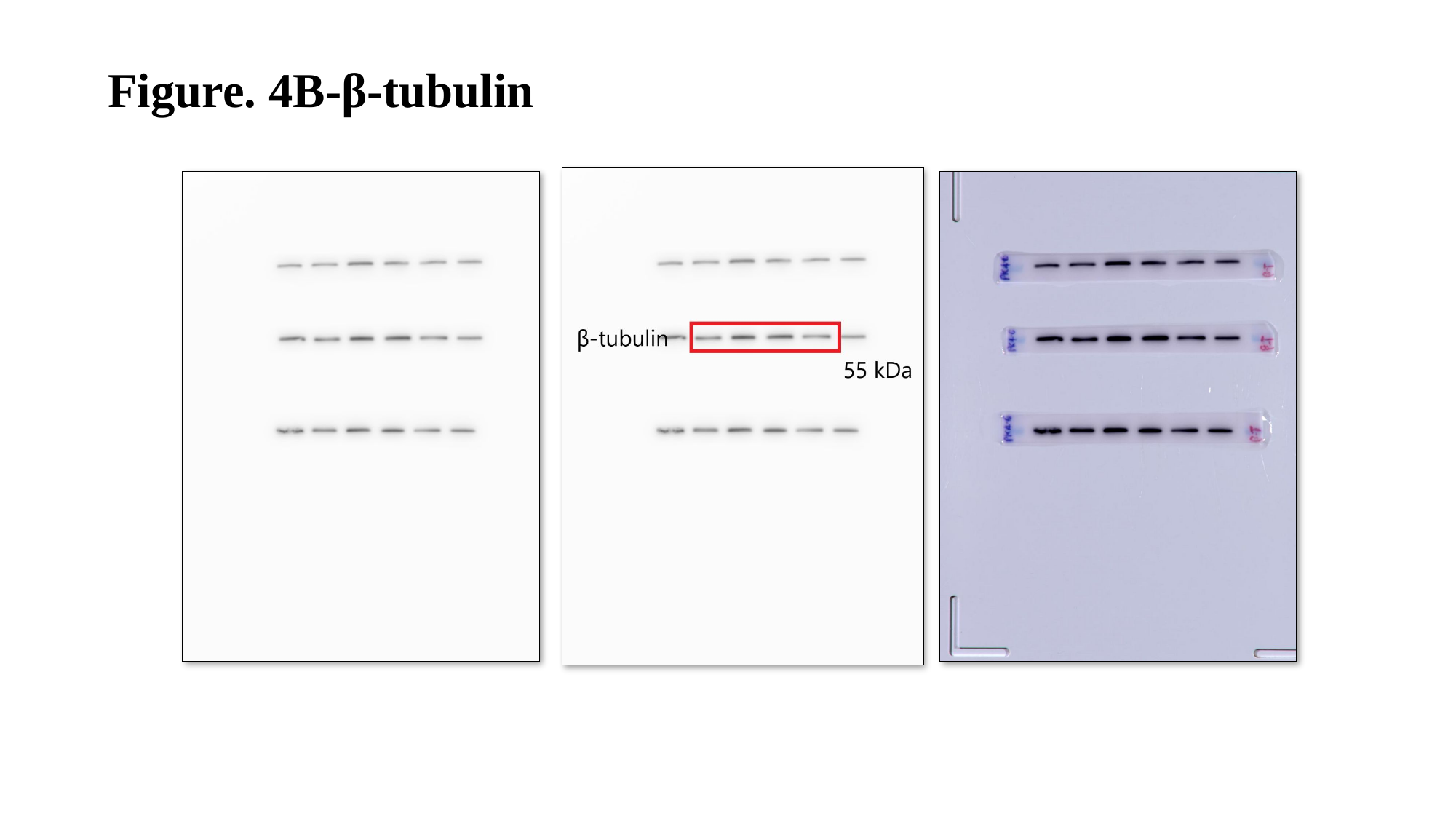

Figure. 4B-β-tubulin

## Slide 16
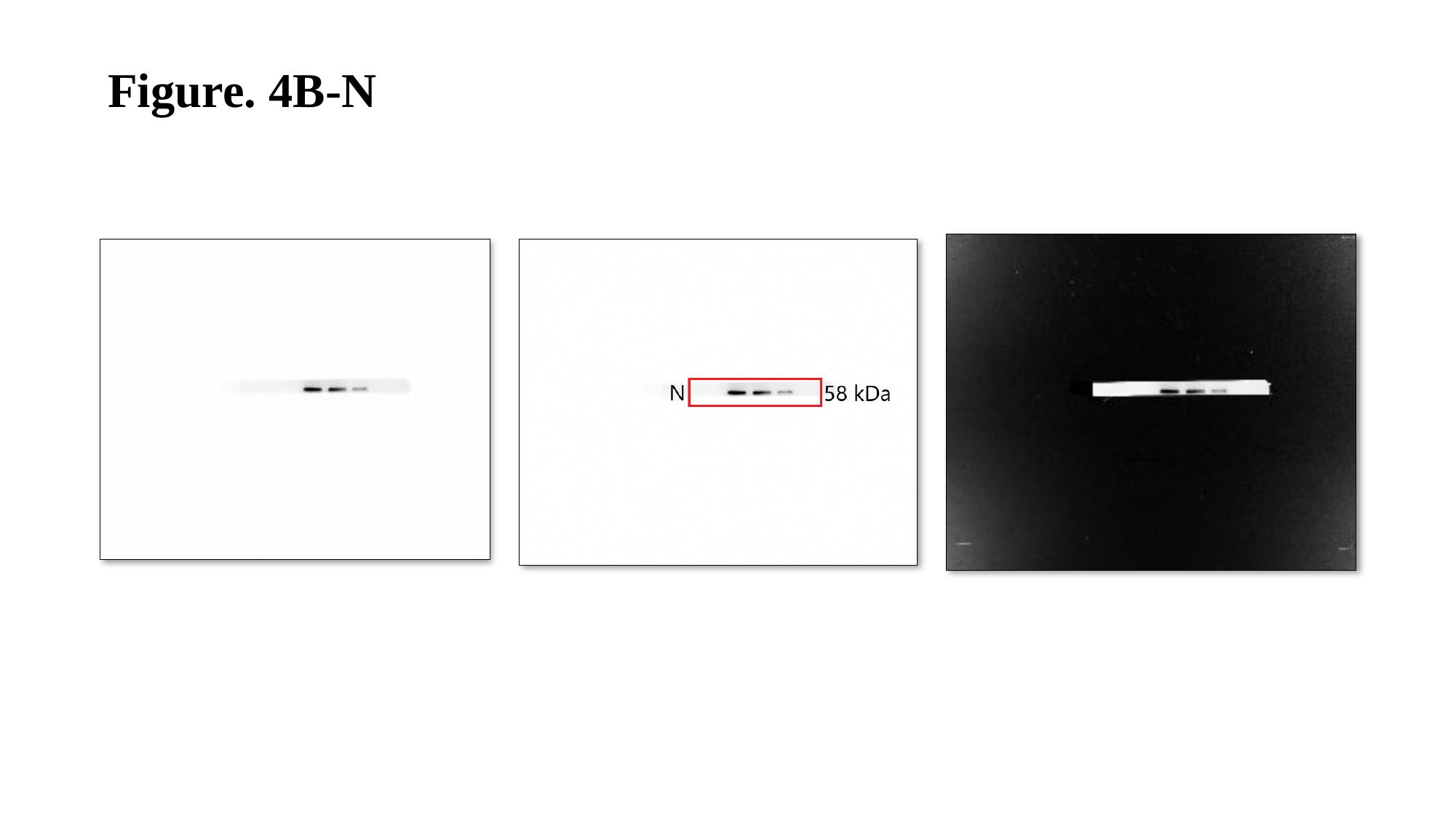

Figure. 4B-N

## Slide 17
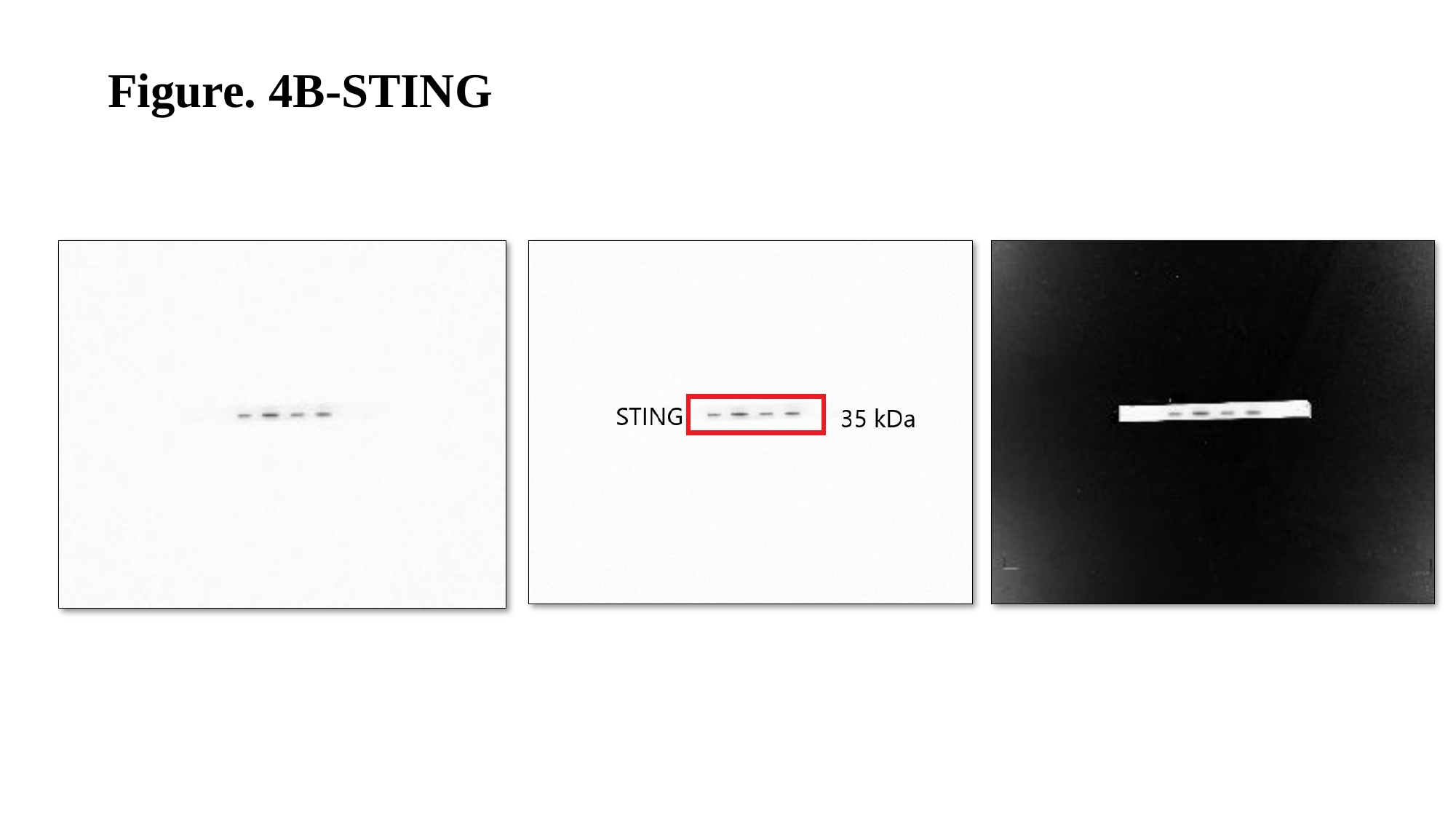

Figure. 4B-STING

## Slide 18
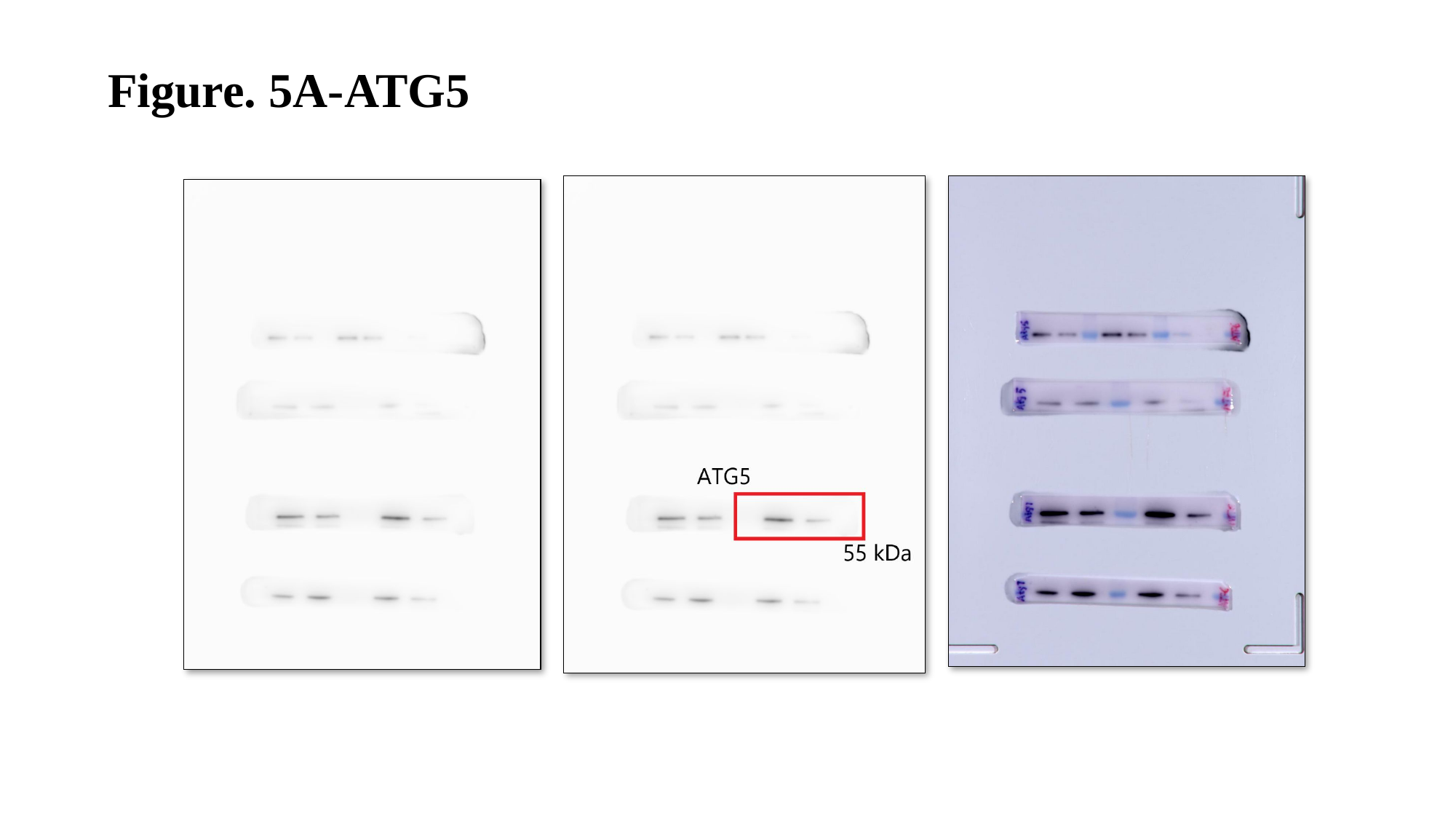

Figure. 5A-ATG5

## Slide 19
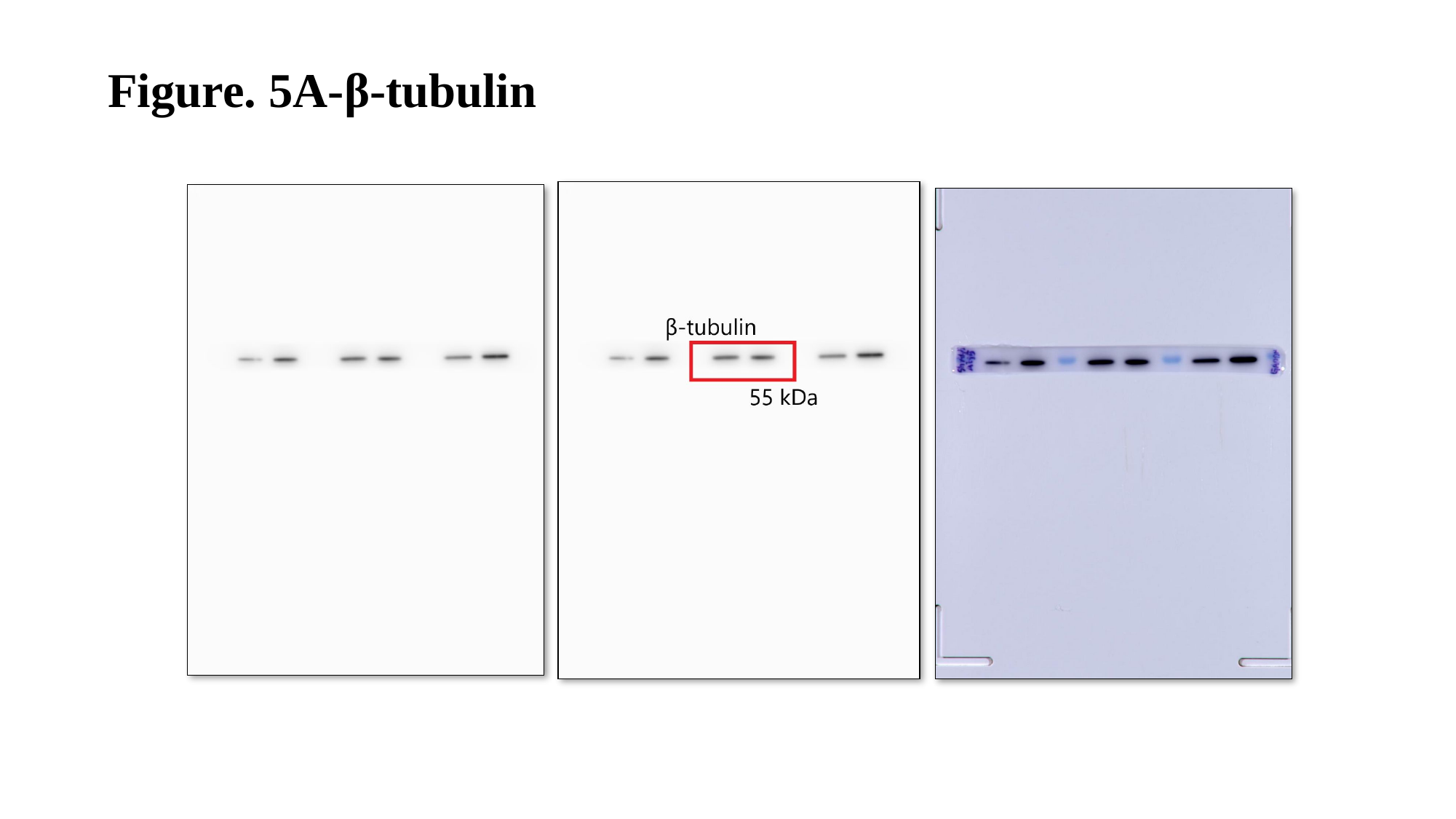

Figure. 5A-β-tubulin

## Slide 20
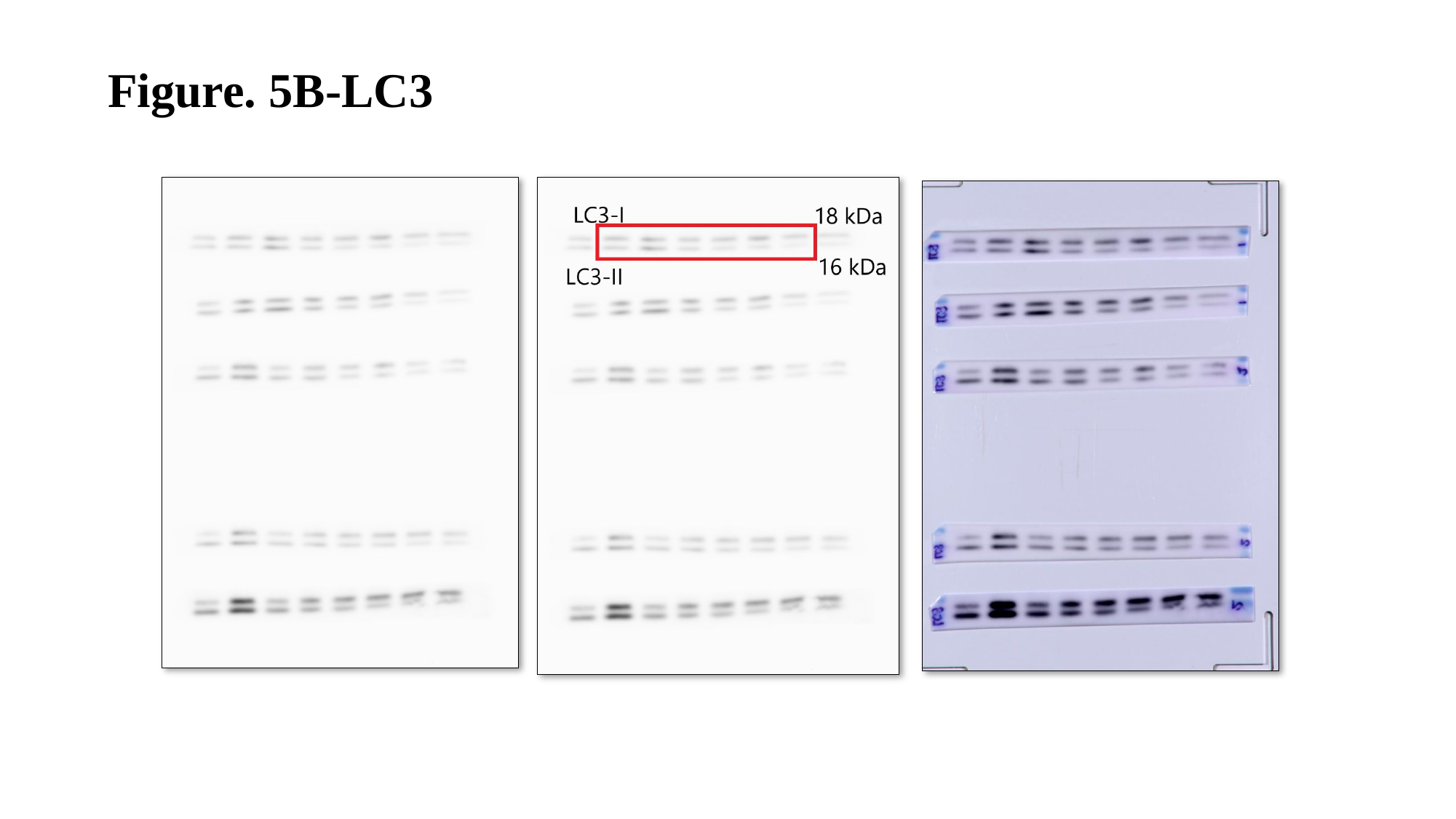

Figure. 5B-LC3

## Slide 21
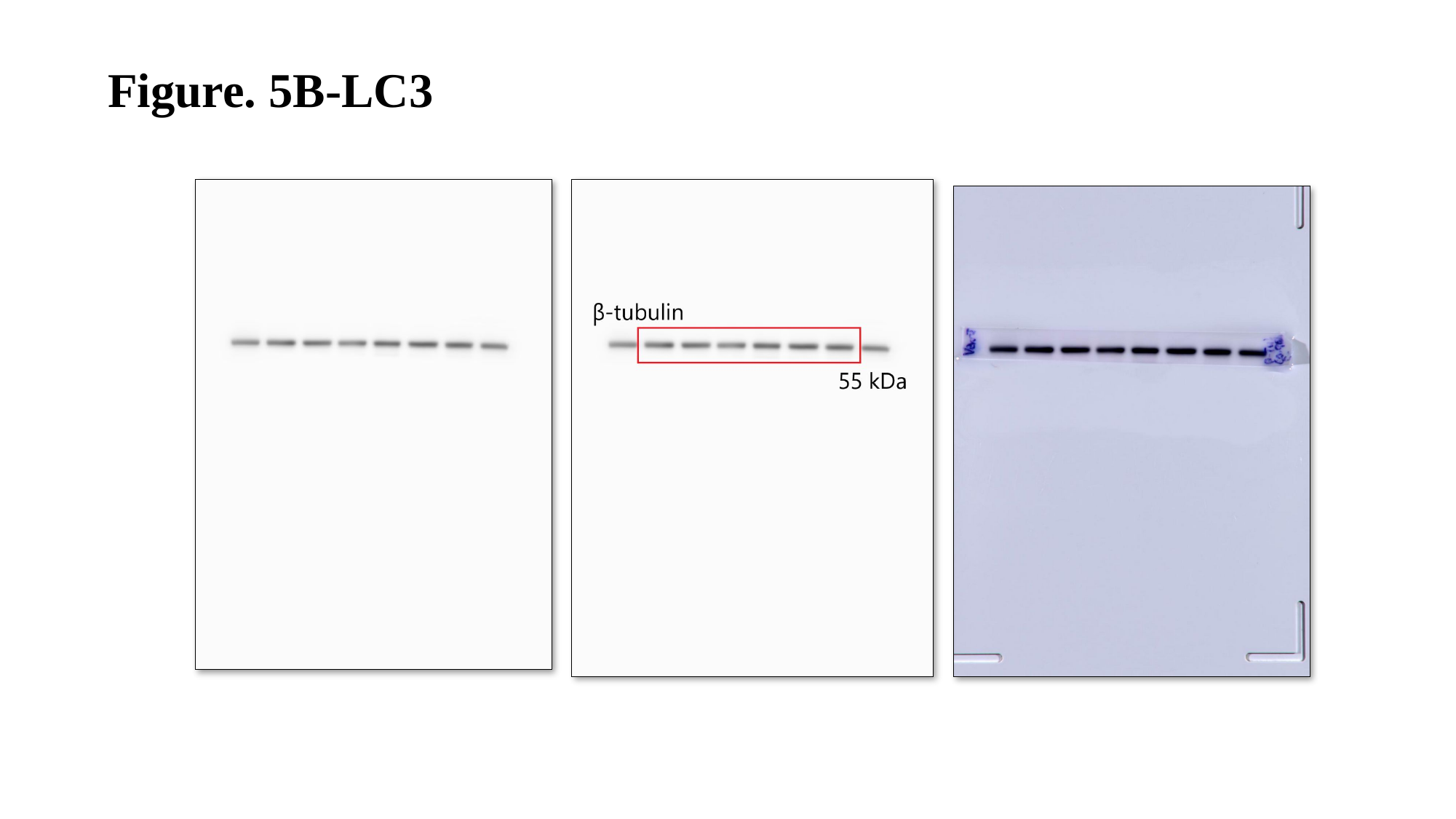

Figure. 5B-LC3

## Slide 22
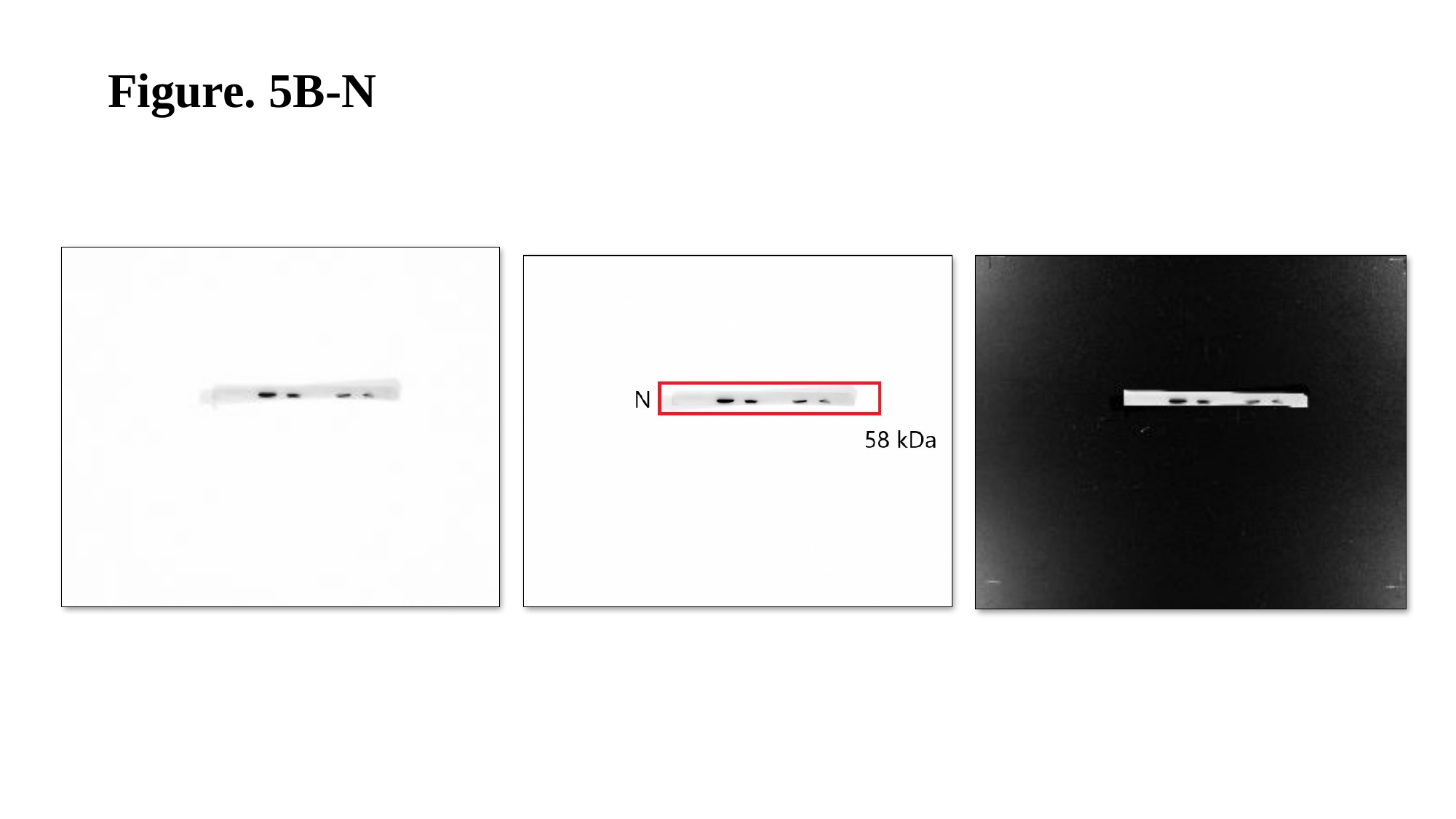

Figure. 5B-N

## Slide 23
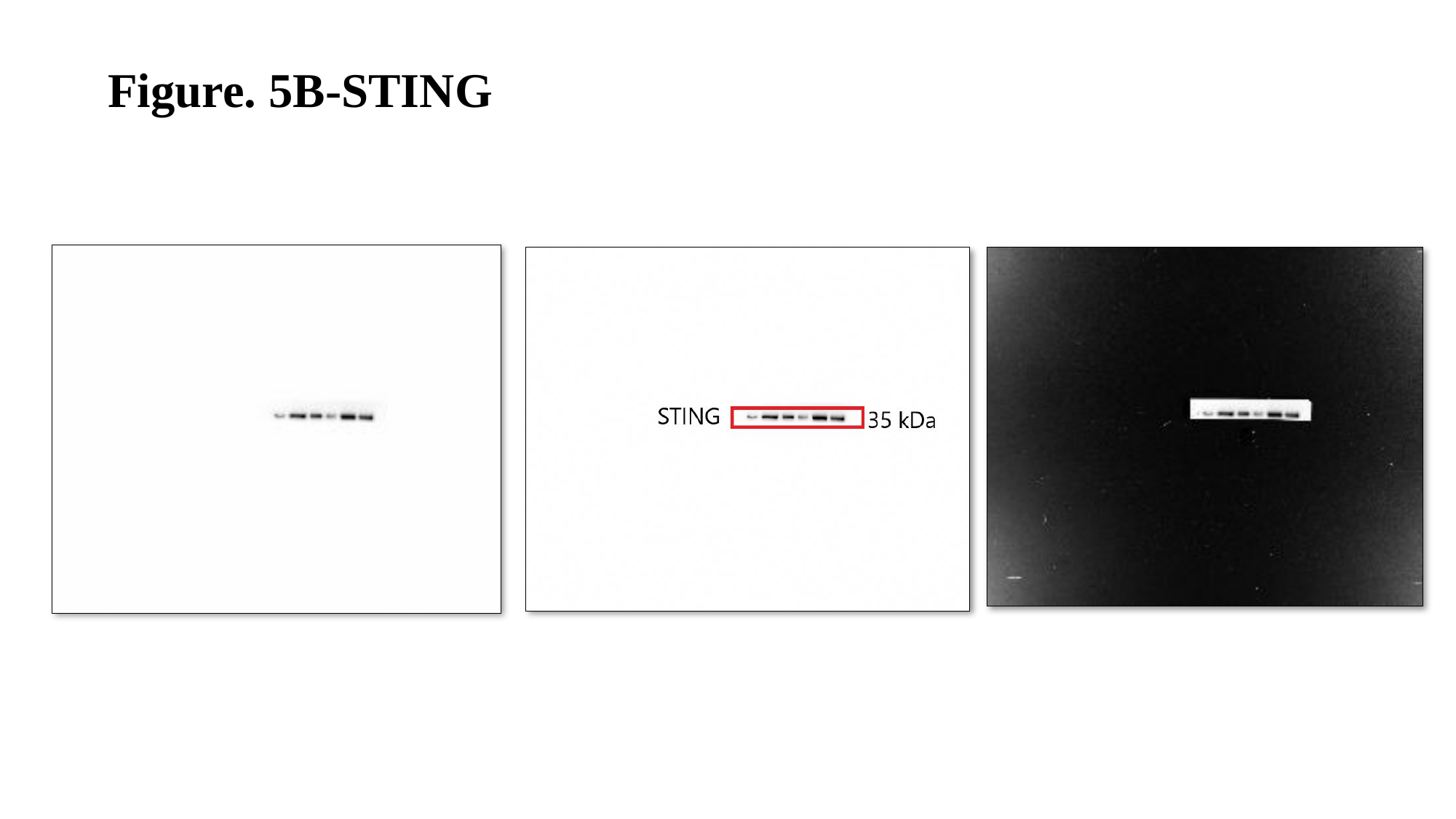

Figure. 5B-STING
